# Phenotypic and Genotypic Characteristics of Members of the Genus *Streptobacillus*

**DOI:** 10.1371/journal.pone.0134312

**Published:** 2015-08-07

**Authors:** Tobias Eisenberg, Werner Nicklas, Norman Mauder, Jörg Rau, Matthias Contzen, Torsten Semmler, Nicola Hofmann, Khayrieh Aledelbi, Christa Ewers

**Affiliations:** 1 Hessian State Laboratory, Department of Veterinary Medicine, Giessen, Germany; 2 German Cancer Research Center, Heidelberg, Germany; 3 Chemical and Veterinary Investigation Office (CVUA) Stuttgart, Fellbach, Germany; 4 Robert Koch Institute, Berlin, Germany; 5 Institute for Multiphase Processes, Leibniz University, Hannover, Germany; 6 Merlin Diagnostika, Bornheim-Hersel, Germany; 7 Institute of Hygiene and Infectious Diseases of Animals, Giessen, Germany; Belgian Nuclear Research Centre SCK•CEN, BELGIUM

## Abstract

The genus *Streptobacillus* (*S*.) remained monotypic for almost 90 years until two new species were recently described. The type species, *S*. *moniliformis*, is one of the two etiological agents of rat bite fever, an under-diagnosed, worldwide occurring zoonosis. In a polyphasic approach field isolates and reference strains of *S*. *moniliformis*, *S*. *hongkongensis*, *S*. *felis* as well as divergent isolates were characterized by comparison of molecular data (n = 29) and from the majority also by their physiological as well as proteomic properties (n = 22). Based on growth-independent physiological profiling using VITEK2-compact, API ZYM and the Micronaut system fastidious growth-related difficulties could be overcome and streptobacilli could definitively be typed despite generally few differences. While differing in their isolation sites and dates, *S*. *moniliformis* isolates were found to possess almost identical spectra in matrix-assisted laser desorption ionization—time of flight mass spectrometry and Fourier transform infrared spectroscopy. Spectroscopic methods facilitated differentiation of *S*. *moniliformis*, *S*. *hongkongensis* and *S*. *felis* as well as one divergent isolate. Sequencing of 16S rRNA gene as well as functional genes *groEL*, *recA* and *gyrB* revealed only little intraspecific variability, but generally proved suitable for interspecies discrimination between all three taxa and two groups of divergent isolates.

## Introduction

For almost 90 years, the genus *Streptobacillus* [1] (*S*.; *Leptotrichiaceae*, *Fusobacteriales*) comprised the monotypic *S*. *moniliformis*, one of the two etiological organisms of rat bite fever (RBF) [2, 3]. Beside RBF *S*. *moniliformis* causes also Haverhill fever (HF), which represent two rarely observed syndromes of this worldwide occurring bacterial zoonosis [3]. The infection is predominantly transmitted through rat bites, scratches or by direct or indirect contact with rat urine [4–6]. Approximately 50–100% of wild rats carry *S*. *moniliformis* in their oro- or nasopharynx and shed the organism with saliva and urine [2, 7, 8]. Despite its wide distribution in natural hosts *S*. *moniliformis* is a rarely detected and most likely under-reported pathogen in humans and animals [3]. In the last few years *Streptobacillus*-like organisms have been observed beside *S*. *moniliformis*, from which *S*. *hongkongensis* [9] and *S*. *felis* [10] were recently described as new species causing quinsy and septic arthritis in humans and pneumonia in a cat, respectively. Furthermore, *Streptobacillus* spp. were reported from a canine oral microbiome project (sequence COT-370; [11]) as well as from Japanese rats [12]. The present study aimed to compare 29 field isolates and reference strains of *S*. *moniliformis*, *S*. *hongkongensis*, *S*. *felis* as well as two yet undescribed species from various geographic areas, host species and isolation sites with respect to phenotypic and molecular properties. In contrast to earlier investigations [13–15] a spectrum of methods was employed to overcome known diagnostic difficulties due to the fastidious growth of this microorganism.

## Materials and Methods

### Bacterial strains

The present study included 29 members of the genus *Streptobacillus* from different host species which cover isolations of *S*. *moniliformis* over the past 90 years from La Réunion near Africa, Asia, Australia, Europe and North America as well as type strains of *S*. *moniliformis*, *S*. *hongkongensis* and *S*. *felis* (Table 1). Eight field isolates and reference strains (“strains” hereafter) were associated with human infections; 17 were obtained from rodents, i.e. 12 originated from rats, four from mice and one strain was derived from a spinifex hopping mouse (*Notomys alexis*); three strains were isolated from turkeys and one strain was isolated from a cat. From seven Japanese rat strains there was only DNA available. The *S*. *moniliformis* strains associated with human disease were isolated from cases of RBF (n = 5), HF (n = 1) and unknown origin (n = 1) (Table 1).

**Table 1:** Field isolates and reference strains as well as origins, clinical symptoms and host species of *Streptobacillus moniliformis*, *Streptobacillus* spp. and *Sebaldella termitidis* used in this study.

Strain no.	Strain designation	Species	Year of isolation	Host	Clinic / sample	Country	Strain reference
1	DSM 12112^T^ (= ATCC 14647^T^)	*S*. *moniliformis*	1925	Human	RBF	France	[1]
2	CIP 55–48	*S*. *moniliformis*	1947	Mouse	Lymph adenitis	UK	
3	ATCC 27747	*S*. *moniliformis*	1964	Turkey	Septic arthritis	USA	[16]
4	NCTC 10773	*S*. *moniliformis*	1971	Human	Blood culture	UK	
5	NCTC 11194	*S*. *moniliformis*	1977	Human	RBF	UK	
6	AHL 370–1	*Streptobacillus* sp.	1979	Spinifex hopping mouse	Liver	Australia	[17]
7	IPDH 144/80	*S*. *moniliformis*	1980	Turkey	Septic arthritis	Germany	
8	CIP 81–99	*S*. *moniliformis*	1981	Human	Blood culture (wild rat bite)	France	
9	AHL 370–4	*S*. *moniliformis*	1982	Mouse	Ear infection	Australia	
10	NCTC 11941	*S*. *moniliformis*	1983	Human	Haverhill fever	UK	
11	IPDH 109/83	*S*. *moniliformis*	1983	Turkey	Septic arthritis	Germany	
12	ATCC 49567	*S*. *moniliformis*	1989	Mouse	Lymph adenitis	Germany	[18]
13	Kun 3 (RIVM)	*S*. *moniliformis*	1991	Rat	Healthy	The Netherlands	[19]
14	ATCC 49940	*S*. *moniliformis*	1992	Rat	Otitis media	Germany	[20]
15	B10/15	*S*. *moniliformis*	unknown	Wild rat	Unknown	The Netherlands	
16	A378/1	*S*. *moniliformis*	1995	Wild rat	Vaginal swab	Germany	DKFZ strain collection
17	VA11257/2007	*S*. *moniliformis*	2007	Human (farmer)	RBF, endocarditis	Germany	[21]
18	VK105/14	*S*. *moniliformis*	2008	Domestic rat	Abscess	Germany	TiHo strain collection
19	B5/1	*S*. *moniliformis*	2009	Laboratory mouse	After rat bite	Germany	DKFZ strain collection
20	Marseille	*S*. *moniliformis*	2009	Rat	RBF	La Réunion	[22]
21	IKC1	*S*. *moniliformis*		Rat	Oral swab	Japan	AB330754, inactivated (DNA); [12]
22	IKC5	*S*. *moniliformis*		Rat	Oral swab	Japan	AB330755, inactivated (DNA); [12]
23	IKB1	*S*. *moniliformis*		Rat	Oral swab	Japan	AB330756, inactivated (DNA); [12]
24	TSD4	*S*. *moniliformis*		Rat	Oral swab	Japan	AB330757, inactivated (DNA); [12]
25	OGS16	*Streptobacillus* sp.		Rat	Oral swab	Japan	AB330758, inactivated (DNA); [12]
26	KWG2	*Streptobacillus* sp.		Rat	Oral swab	Japan	AB330759, inactivated (DNA); [12]
27	KWG24	*Streptobacillus* sp.		Rat	Oral swab	Japan	AB330760, inactivated (DNA); [12]
28	131000547^T^ (DSM 29248^T^)	*S*. *felis*	2013	Cat	Pneumonia	Germany	[10]
29	DSM 26322^T^ (HKU33^T^)	*S*. *hongkongensis*	2014	Human	Abscess	Hong Kong	[9]
30	NCTC 11300^T^ (ATCC 33386^T^)	*Sebaldella termitidis*	1962	Termite	Intestine		

^T^: type strain; ATCC: American Type Culture Collection, Rockville, USA; NCTC: National Collection of Type Cultures, London, UK; CIP: Collection Institut Pasteur, Paris, France; IPDH: Institut für Geflügelkrankheiten, Hannover, Germany; RIVM: Rijksinstituut voor Volksgezondheid en Milieuhygiene, Bilthoven, The Netherlands; AHL: Animal Health Laboratory, South Perth, Australia; ZfV: Zentralinstitut für Versuchstierzucht, Hannover, Germany; DKFZ: Deutsches Krebsforschungszentrum, Heidelberg, Germany; TiHo: Tierärztliche Hochschule Hannover, Germany; RBF: rat bite fever

### Phenotypic characterization

#### Culture requirements

For the growth of *Streptobacillus* spp. Columbia agar supplemented with 5% sheep blood (Oxoid, Wesel, Germany; SBA) was incubated for 2–5 days at 37°C in a capnophilic atmosphere of 10% CO_2_. Liquid media (brain-heart infusion and peptone broth, supplemented with 20% bovine or horse serum [all Oxoid]) were used for streptobacillary growth and incubated for 2–7 days under the same conditions.

#### Biochemical properties

Extended biochemical profiling was carried out according to the manufacturer’s instructions using commercial fermentative test systems, i.e. Micronaut Strep2 and RPO (Merlin Diagnostika, Bornheim-Hersel, Germany; [23]), VITEK2-compact with the NHI card and API ZYM (both bioMeriéux, Nürtingen, Germany; Table 2).

**Table 2:** Physiological characteristics of field isolates and reference strains from *Streptobacillus moniliformis* and of reference strains from *Streptobacillus felis* 131000547^T^, *Streptobacillus hongkongensis* DSM 26322^T^ and *Sebaldella termitidis* NCTC 11300^T^.

	Strain no.																						
Compound	1	2	3	4	5	6	7	8	9	10	11	12	13	14	15	16	17	18	19	20	28	29	30
haemolysis on SBA	-	-	-	-	-	-	-	-	-	-	-	-	-	+	-	-	-	-	-	-	+	+	-
tripeptidase* ^#^	+	+	+	+	+	+	+	+	+	+	-	+	+	+	+	+	+	+	+	+	+	-	+
prolin aminopeptidase* ^#^	+	+	+	+	+	+	+	+	+	+	-	+	+	+	+	+	+	+	+	+	+	-	-
hydroxyprolin aminopeptidase* ^#^	+	+	+	+	+	+	+	+	+	+	-	+	+	+	+	+	+	+	+	+	+	-	-
glycyltryptophan aminopeptidase* ^#^	+	+	+	+	+	+	+	+	+	+	+	+	+	+	+	+	+	+	+	+	+	**-** ^§^	-
arginine aminopeptidase* ^#^	+	+	+	+	+	+	+	+	+	+	+	+	+	+	+	+	+	-	+	-	+	-	-
pyrase* ^#^	+	+	+	+	+	+	+	+	+	+	+	+	+	+	+	+	+	+	+	+	+	**-** ^§^	-
neuraminidase* ^#^	+	+	+	+	+	+	+	+	+	+	+	+	+	-	+	w	+	-	+	+	-	-	-
arginine dihydrolase* ^#^	+	+	+	+	+	+	+	+	-	+	-	+	+	+	+	-	+	+	+	+	-	+	-
glycylprolin aminopeptidase* ^#^	+	+	+	+	+	+	+	+	+	+	+	+	+	+	+	+	+	+	+	+	+	+	+
asparatyl aminopeptidase* ^#^	-	-	-	-	-	-	-	-	-	-	-	-	-	-	-	-	-	-	-	-	-	-	-
growth in SPMS medium* ^#^	+	+	+	+	+	+	+	+	+	+	+	+	+	+	+	+	+	+	+	+	+	+	-
urease*	-	-	-	-	-	-	-	-	-	-	+	-	-	-	-	-	+	-	-	-	+	-	-
p-nitrophenyl-β-D-glucuronide*	-	-	-	-	-	-	-	-	-	+	-	-	-	-	-	-	-	-	-	-	-	-	-
esculine*	-	-	-	-	-	-	-	-	-	-	-	-	-	-	+	-	-	-	-	-	-	-	+
phenylalanine arylamidase^†^	+	+	+	+	+	+	+	+	+	+	+	+	+	+	+	+	+	+	+	+	**-** ^§^	**-** ^§^	-
L-pyrrolidonyl arylamidase^†^	+	-	-	+	-	-	+	+	+	+	+	+	+	-	+	+	+	-	+	+	-	-	-
phospahatase^†^	-	-	-	-	-	-	-	-	-	-	-	-	-	-	-	-	-	-	-	-	-	**+** ^§^	-
tyrosine arylamidase^†^	+	+	+	+	+	w	+	+	+	+	+	+	+	+	+	+	+	w	+	+	-^§^	-^§^	-
ala-phe-pro-arylamidase^†^	+	+	+	+	+	+	+	+	+	+	+	+	+	+	+	+	+	+	+	+	-^§^	-^§^	+
phenylphosphonate^†^	-	w	-	-	-	-	-	-	-	-	-	-	-	-	-	-	-	-	-	-	-	-	+
D-mannose^†^	-	+	-	+	-	-	-	-	-	-	-	-	-	-	-	-	-	-	-	-	-	-	+
N-acetyl-D-glucosamine^†^	-	-	-	-	-	-	-	-	-	-	-	-	-	-	-	-	-	w	-	-	-	+	+
D-glucose^†^	+	w	-	+	-	-	-	-	-	+	-	-	-	-	-	-	+	-	-	+	-	+	+
alkaline phosphatase^‡^	w	-	w	w	-	-	w	-	-	+	-	w	w	-	-	-	-	-	-	-	+	+	+
esterase (C4)^‡^	w	w	w	w	w	+	w	-	w	+	w	w	+	w	-	w	-	-	-	-	+	w	-
esterase lipase (C8)^‡^	+	+	+	+	+	+	w	w	+	+	+	+	+	+	w	+	w	w	w	w	+	w	-
lipase (C14)^‡^	-	-	-	-	-	-	-	-	-	-	-	-	-	-	-	-	-	-	-	-	-	-	-
leucine arylamidase^‡^	w	-	w	w	-	w	w	w	-	w	w	+	-	-	-	-	-	-	-	-	-	-	-
valine arylamidase^‡^	-	-	-	-	-	-	-	-	-	-	-	-	-	-	-	-	-	-	-	-	-	-	-
cystine arylamidase^‡^	-	-	-	-	-	-	-	-	-	-	-	-	-	-	-	-	-	-	-	-	-	-	-
trypsin^‡^	-	-	-	-	-	-	-	-	-	-	-	-	-	-	-	-	-	-	-	-	-	-	-
α-chymotrypsin^‡^	+	w	w	+	+	+	-	w	w	w	w	+	+	w	+	+	+	+	+	+	-	-	-
acid phosphatase^‡^	w	-	-	-	-	+	w	-	-	-	-	-	-	-	-	-	-	-	-	-	+	+	+
naphthol-AS-BI-phosphohydrolase^‡^	-	-	-	-	-	-	-	-	-	-	-	-	-	-	-	-	-	-	-	-	-	w	w
β-Glucuronidase^‡^	-	-	-	-	-	-	-	-	-	-	-	-	-	-	-	-	-	-	-	-	-	-	-
α-Glucosidase^‡^	-	-	-	-	-	-	-	-	-	-	-	-	-	-	-	-	-	-	-	-	-	-	-

Physiological characteristics were obtained by an individual panel of eleven^#^ discriminatory reactions designed for the identification of *Streptobacillus* spp. (Micronaut Strep2 and RPO; all Merlin Diagnostika GmbH)*, VITEK2-compact with the NHI card^†^, API-ZYM^‡^ (both bioMeriéux) and haemolytic properties on Columbia agar with 5% sheep blood; for congruent results see text; +: positive;-: negative; w: weak reaction;

^§^ potential discriminatory character for species identification

Presumptive physiological characterization further employed standard microbiological procedures: Haemolytic properties of the bacteria were observed on SBA. Tests for catalase activity were carried out with 3% H_2_O_2_ on microscopic slides and those for cytochrome oxidase with the BBL DrySlide system (Becton Dickinson, Heidelberg, Germany).

#### Antimicrobial susceptibility testing

The antimicrobial susceptibility pattern was determined using minimal inhibitory concentrations (MIC) obtained by broth microdilution test (Merlin Diagnostika) as described earlier [24]. Following the adaptation of the read-out system by using SPMS culture medium containing cattle serum and gelatine, the commercially available Micronaut-S *Campylobacter* (all Merlin Diagnostika) was carried out (S1 Table). The test design contained the following 12 antimicrobial substances: azithromycin (AZM), ciprofloxacin (CIP), clindamycin (CLI), chloramphenicol (CMP), erythromycin (ERY), gentamicin (GEN), meropenem (MER), nalidixic acid (NAL), streptomycin (STR), trimethoprim/sulfamethoxazol (T/S), telithromycin (TEL), and tetracycline (TET). Results were interpreted according to Clinical and Laboratory Standards Institute criteria [25].

#### Haemagglutination

Screening for adhesive properties was performed for 15 strains (No. 1–15 according to Table 1) by previously described haemagglutination experiments using erythrocytes from 11 different host species [26]. In detail, red blood cells from humans (blood type AB, rhesus factor positive), BALB/c and C57B1/6J mice, rats, turkeys, guinea-pigs, hamsters, chickens, sheep, horses, pigs and cattle were included [26]. For slide agglutination experiments defibrinated blood samples were diluted 1:4 in phosphate buffered saline (PBS). Colonies from a 24 h culture of *S*. *moniliformis* were used in a turbidity of McFarland 6 in 150 μl PBS. Strong reactions were read as agglutination after gently mixing 15 μl bacterial suspensions with 10 μl diluted blood samples after 30 sec. Delayed reactions were read after 2 min incubation on ice. Haemagglutination was also assessed in microtiter plates by adding the bacterial suspension from the slide agglutination experiment to a final volume of 60 μl per well thereby yielding blood dilutions of 1:7 and 1:10. Sealed plates were incubated for 24 h at 4°C. Haemagglutination was detected by semiquantitative reading as strong (++) or moderate/weak (+) shape of coat-forming layers on the well wall, whereas negative reactions caused sedimentation of erythrocytes on the bottom. *E*. *coli* KK 158/1 and a setup without bacteria served as positive and negative controls, respectively [26].

#### Matrix assisted laser desorption ionization–time of flight mass spectrometry (MALDI-TOF MS)

**Fig 1:**
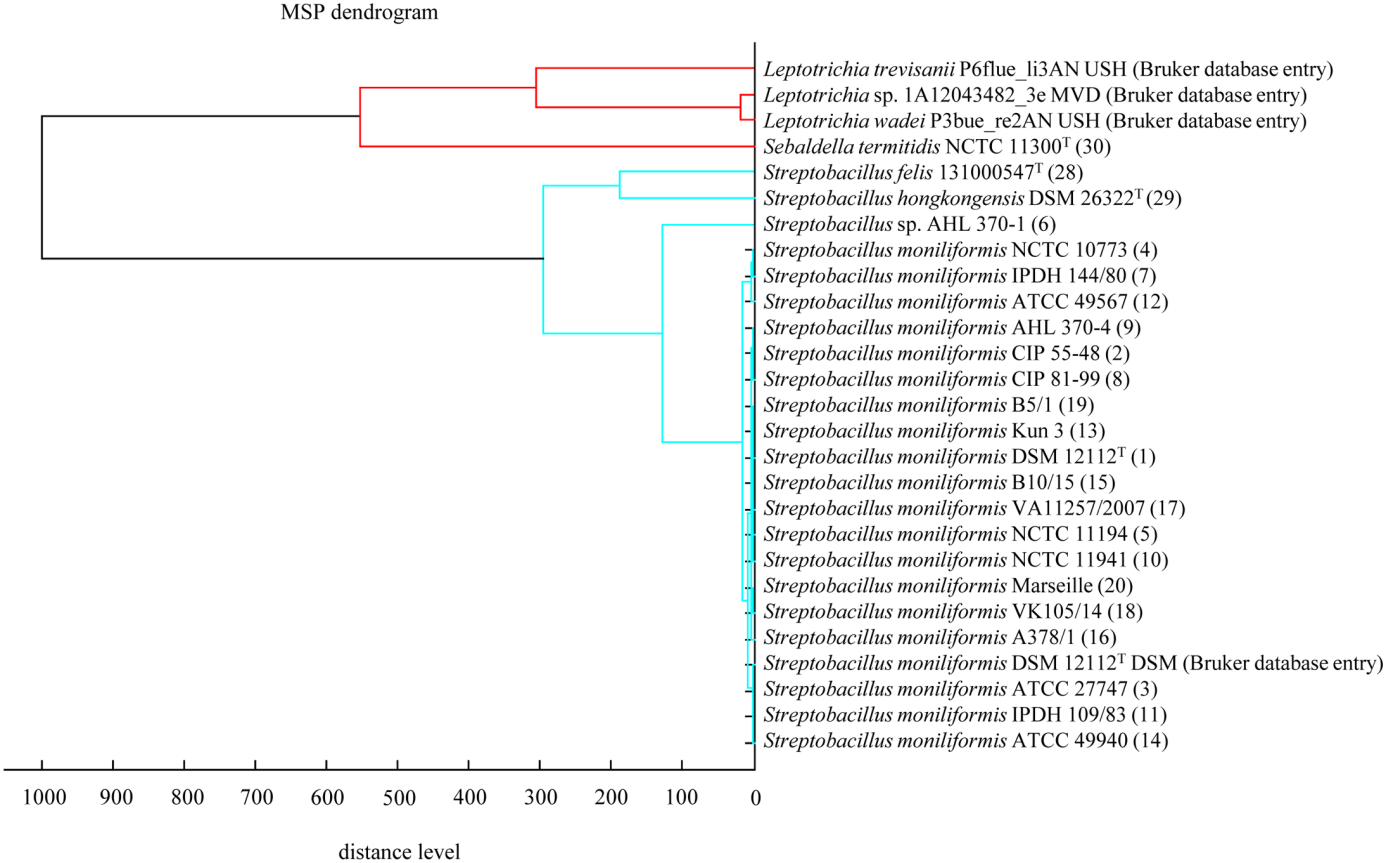
Dendrogram including all main spectra peak lists (MSP) of the family *Leptotrichiaceae* available in the Bruker Taxonomy Database. Spectra of *Streptobacillus moniliformis* field and reference strains, *Streptobacillus hongkongensis* DSM 26322^T^, *Streptobacillus felis* 131000547^T^ and *Sebaldella termitidis* ATCC 33386^T^ were measured using the direct transfer protocol. The dendrogram was generated using the BioTyper MSP Dendrogram Creation Standard Method (v1.4) of the MALDI Biotyper OC Software (v3.1, build 66). The database used (DB 5627, BrukerDaltonics) comprised a singular entry from *S*. *moniliformis* (DSM 12112^T^ = ATCC 14647^T^); ^T^: type strain, ATCC: American Type Culture Collection, Rockville, USA, NCTC: National Collection of Type Cultures, London, UK, DSM: Deutsche Sammlung für Mikroorganismen, Braunschweig, Germany, IPDH: Institut für Geflügelkrankheiten, Hannover, Germany.

Bacteria were incubated for 24 h, subsequently selected from the SBA plates and subjected to steel targets according to manufacturer's instructions (BrukerBiotyper, BrukerDaltonics, Bremen, Germany). The viable bacteria were prepared using the direct smear method as well as an extraction protocol provided by the manufacturer. Briefly, freshly grown bacteria were harvested and diluted in ethanol, centrifuged (2.000 x g, 2 min), air dried and resuspended in aqueous volumes of 70% formic acid and acetonitrile followed by a vortex step. One microliter was directly transferred to the steel target. Analysis was performed on a MALDI-TOF MS Biotyper Version V3.3.1.0. The database used (DB 5627, BrukerDaltonics) comprised only one entry for *S*. *moniliformis* from strain DSM 12112^T^ (= ATCC 14647^T^). A dendrogram including selected main spectra peak lists (MSP) of the family *Leptotrichiaceae* from the Bruker database and the *S*. *moniliformis* strains from this study is depicted in Fig 1.

#### Fourier Transform-Infrared Spectroscopy (FT-IR)

**Fig 2:**
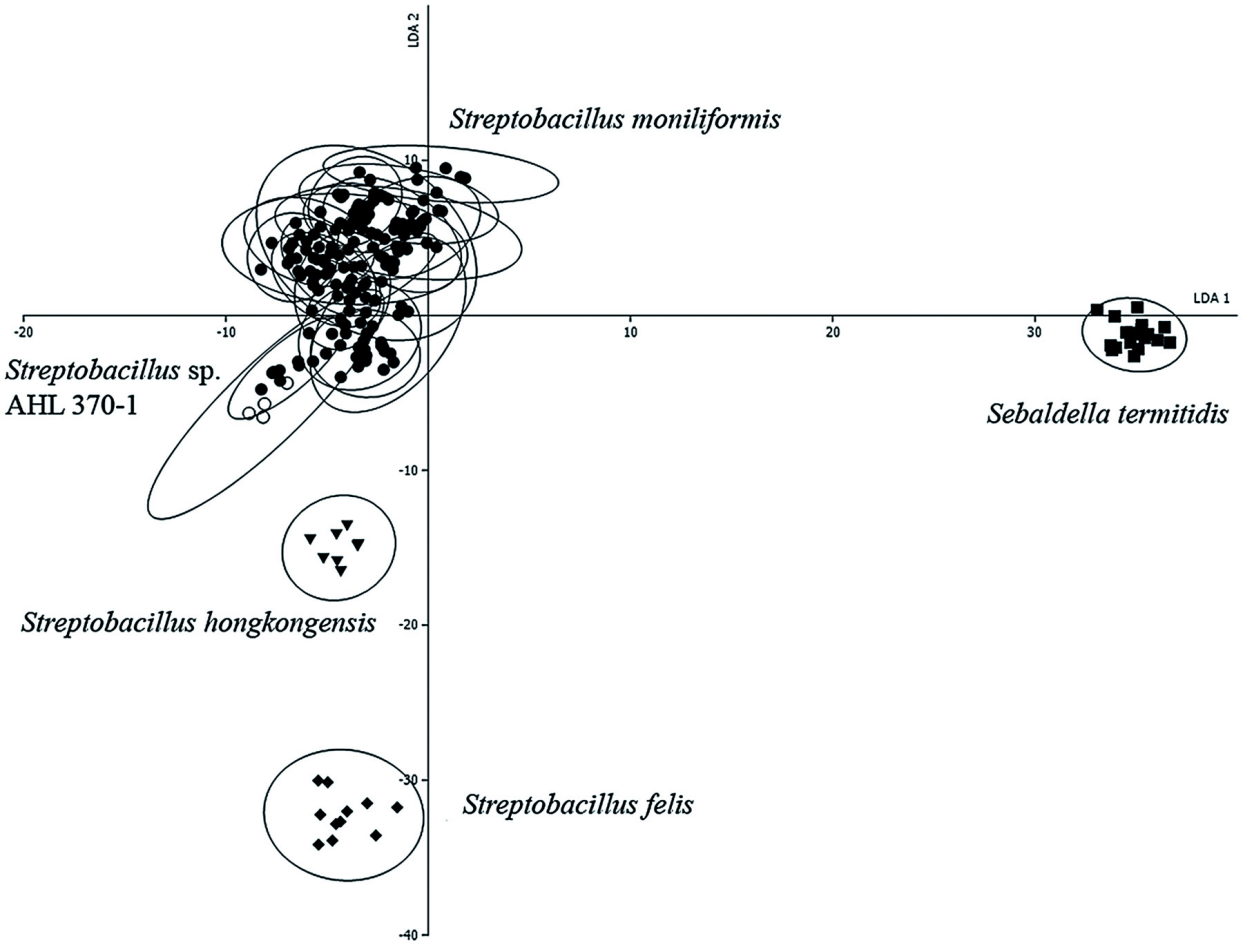
Linear discriminant analysis (LDA) of 201 infrared spectra of one strain each of *Sebaldella termitidis* NCTC 11300^T^, *Streptobacillus hongkongensis* DSM 26322^T^, *Streptobacillus felis* 131000547^T^, 19 *Streptobacillus moniliformis* field isolates and reference strains and *Streptobacillus* sp. isolate AHL 370–1 from a spinifex hopping mouse. The wave numbers 550–1800 cm^-1^ and 2800–3200 cm^-1^ of second derivative spectra were selected and vector normalized. After a principal component analysis, the first 40 components were used for the LDA. In this LDA every isolate was defined as one group. Spectra of *Sebaldella termitidis* ATCC 33386^T^ are represented by squares, *Streptobacillus felis* 131000547^T^ by diamonds, *Streptobacillus hongkongensis* DSM 26322^T^ by triangles, *Streptobacillus* sp. (AHL 370–1) by circles and *Streptobacillus moniliformis* field isolates and reference strains by dots. Ellipses contain 95% of all group spectra assuming a bivariate normal distribution.

Bacterial isolates were cultivated independently in 5–7 replicates at 37°C for five days in a capnophilic atmosphere of 10% CO_2_ on SBA. Harvesting of cells, preparation of bacterial films on zinc selenide plates, drying and handling was performed as described previously [27]. The dried bacterial films were used directly for examination by FT-IR. Infrared spectra were recorded for each sample in a transmission mode from 500 to 4000 cm^-1^ with an FT-IR spectrometer (Tensor27 with HTS-XT-module, BrukerOptics, Ettlingen, Germany). Acquisition and first analysis of data was carried out using OPUS Software (vers. 4.2, BrukerOptics). To get a first impression the IR spectra of all viable strains listed in Table 1 were compared by hierarchical cluster analysis [28, 29]. Therefore, the second derivation of the vector normalized spectra in the wave number range of 500–1400 cm^-1^ and 2800–3000 cm^-1^ were used for calculation with Ward’s algorithm (OPUS 4.2; [30]). Spectra identified as outliers were quashed. The wave numbers 550–1800 cm^-1^ and 2800–3200 cm^-1^ of second derivative spectra were selected and vector normalized. After a principal component analysis, the first 40 components were used for linear discriminant analysis with spectra grouped by isolate. The diagram obtained depicts the arrangement of isolates according to their spectral differences (Fig 2).

### Molecular characterization

#### Species specific PCR for *S*. *moniliformis*

Two PCRs for the detection of *S*. *moniliformis* based on the 16S rRNA gene were performed with minor modifications [12, 31]. Freshly cultured colonies were suspended in 180 μl sterile distilled water. After incubation for 15 min at 100°C the lysates were centrifuged at 12.000 x *g* for 10 min to remove cell debris. One microliter of the supernatant was used for subsequent PCR analysis according to Kimura *et al*. (primers S5: 5’-CATACTCGGAATAAGATGG-3’ and AS2: 5’-GCTTAGCTCCTCTTTGTAC-3’; PCR program as follows: x1 (95°C, 180 sec), x35 (95°C, 20 sec, 53°C, 60 sec, 72°C, 60 sec), x1 (72°C, 420 sec)) [12] and Nicklas (primers SbmF: 5’-GAGAGAGCTTTGCATCCT-3’ and SbmR: 5’-GTAACTTCAGGTGCAACT-3’; x1 (94°C, 240 sec), x35 (94°C, 60 sec; 50°C, 60 sec; 72°C, 60 sec), x1 (72°C, 420 sec)) (cited in [31]), respectively. All PCRs were carried out in a T3000 Thermocycler (Biometra, Göttingen, Germany). Amplicons of 269 and 1222 bp, respectively, were detected by electrophoresis in 1.5% agarose gel containing ethidium bromide (1 mg/ml), visualised in an UV transilluminator and photographed. DNA extracted from *S*. *moniliformis* strain NCTC 11941 served as positive control in all runs.

#### DNA sequence analysis and determination of guanine/cytosine (G/C) contents

DNA was extracted from a pure bacterial culture from all viable strains with a commercial kit according to the manufacturer’s instructions (MasterPure Complete DNA and RNA Purification Kit, Epicentre, distributed by Biozym Scientific, Hessisch Oldendorf, Germany) and DNA from all strains was subjected to genome sequencing. De novo assembly was performed with CLC Genomics Workbench, Version 7.5 (CLC Bio, Aarhus, Denmark). For automatic annotation we used the RAST Server: Rapid Annotations using Subsystems Technology [32]. Sequence analysis from non-published genomes and calculation of G/C contents were carried out with Geneious (v. 8.1.3; Biomatters, Auckland, NZ) [33]. Nucleotide sequences of partial 16S rRNA genes and of *groEL*, *recA* and *gyrB* genes were aligned by using MAFFT [34] (MAFFT v7.017, implemented in Geneious). Maximum likelihood phylogenies and trees were estimated (100 bootstrap replicates) and visualized with PhyML [35], using the HKY85 model [36].

Instead of weak DNA-DNA hybridization results for members of this genus [26] (data not shown) average nucleotide identity (ANI) was carried out according to the method described by Goris et al. [37] using the ezbiocloud platform (http://www.ezbiocloud.net/ezgenome/ani). According to Richter & Rosello-Mora [38] the cut-off for species boundary with this method is at 95–96%.

## Results

### Phenotypic characterization

#### Presumptive confirmation of bacterial strains

Streptobacilli grew well on SBA after 2–5 days of incubation at 37°C in 10% CO_2_. Colonies were tiny, drop-like, shiny, slightly convex, 0.1–0.4 mm in diameter. Some of the colonies showed a “fried-egg” appearance. Strains normally grew without haemolysis, but strains *S*. *moniliformis* ATCC 49940, *S*. *felis* 131000547^T^ and *S*. *hongkongensis* DSM 26322^T^ displayed alpha-haemolytic colonies as already described [10, 20, 39]. In liquid media streptobacillary growth could be detected after 2–7 days as “puff-ball” or “bread crumb-like” appearance. Gram staining revealed irregular Gram-negative pleomorphic, fusiform to filamentous, non-spore forming, non-encapsulated, non-acid-fast rods that were arranged in chains and clumps, sometimes displaying irregular, lateral bulbar swellings.

#### Biochemical studies

Despite its Gram-negative staining characteristics of *S*. *moniliformis*, a representative species specific panel of biochemical reactions was derived from preliminary testing with the Micronaut Strep2 (for streptococci and enterococci) and RPO (for Gram-positive bacteria) test systems. A panel of eleven relevant chemotaxonomic discriminatory parameters proved sufficient for the identification of *S*. *moniliformis*, which included besides principle growth characteristics and negative fermentative reactions during overnight incubation (data not shown) the following reactions (positive percentage): tripeptidase (95.2), proline aminopeptidase (95.2), arginine dihydrolase (85.7), arginine aminopeptidase (90.5), glycyltryptophan aminopeptidase (100), growth in SPMS medium (100), neuraminidase (90.5), glycylproline aminopeptidase (100), hydroxyproline aminopeptidase (95.2), pyrase (100) and asparatyl aminopeptidase (0) (Table 2). Further physiological tests obtained by Micronaut Strep2 and RPO gave congruent results for all *S*. *moniliformis* strains for hydrolysis of p-nitrophenyl-β-D-glucosamide, alkaline phosphatase and arginine hydrolysis (positive) as well as for tellurite, fermentation of cellobiose, arbutin, sorbitole, amygdaline and raffinose, chitin, H-asp-β-naphthylamide, p-nitrophenyl-α-D-galactopyranoside, p-nitrophenyl-β-D-galactopyranoside, p-nitrophenyl-β-D-fucopyranoside, p-nitrophenyl-β-D-glucopyranoside (negative). Neither the single strains of *S*. *felis* 131000547^T^ and *S*. *hongkongensis* DSM 26322^T^ nor strain AHL 370–1 could unambiguously be separated from *S*. *moniliformis* by the above mentioned physiological characters, although *S*. *hongkongensis* was the only glycyltryptophan aminopeptidase- and pyrase-negative strain under study. VITEK2-compact identified strains of *S*. *moniliformis*, *S*. *felis* 131000547^T^ and *S*. *hongkongensis* DSM 26322^T^ as *Neisseria* (*N*.) *cinerea* (93–99% confidence), *N*. *elongata* (87% confidence) or inconclusive between *N*. *cinerea* and *N*. *elongata* (NHI card profiles 0210000002, 0220000000, 0220000040, 0233000000, 0232000000, 0233000000, 0273000000, 0273200000, 0277220000, 0237100002, 0237220000). From the 30 different reactions all but eleven were congruently negative for arginine-arylamidase, γ-glutamyl-transferase, L-lysine-arylamidase, D-galactose, Ellman’s reagent, L-pyrrolidonyl-arylamidase, tyrosine-arylamidase, glycogen, D-maltose, sucrose, urease, β-galactopyranosidase indoxyl, ornithine-decarboxylase, α-arabinosidase, pyruvate, phosphoryl-choline, D-malate, maltotriose, L-glutamine, D-ribose, phenylphosphonate and D-xylose and positive for leucine-arylamidase and L-proline-arylamidase for all tested 22 strains of the genus *Streptobacillus* (nos. 1–20, 28, 29 according to Table 2). All *S*. *moniliformis* strains were phenylalanine-arylamidase-, tyrosine-arylamidase- and ala-phe-pro-arylamidase-positive in contrast to *S*. *felis* 131000547^T^ and *S*. *hongkongensis* DSM 26322^T^. Furthermore, *S*. *hongkongensis* DSM 26322^T^ was the only phosphatase-positive strain under investigation.

The API-ZYM test was carried out with 22 *Streptobacillus* strains and revealed consistent enzymatic pattern for lipase (C14), valine arylamidase, cystine arylamidase, trypsin, α-glucosidase, α-galactosidase, β-galactosidase, β-glucuronidase, β-glucosaminidase, N-acetyl-β-glucosaminidase, α-mannosidase, α-fucosidase (all negative). All *Streptobacillus* strains from this study were positive for esterase lipase (C8). Contrarily, *S*. *hongkongensis* DSM 26322^T^ was the only naphthol-AS-BI-phosphohydrolase-positive strain and strain IPDH 144/80 was solely α-chymotrypsin-negative. All further differing biochemical test results are presented in Table 2. Presumptive physiological characterization revealed corresponding results for all strains for cytochrome oxidase, catalase, urease, nitrate reduction and indole production (all negative).

#### Antimicrobial susceptibility testing

Antimicrobial susceptibility was determined for all 22 viable *Streptobacillus* strains tested in this study and results are presented in S1 Table. Congruent *in vitro* results could be obtained for all strains with respect to azithromycin (≤0.0625–2), clindamycin (≤0.125–0.25), chloramphenicol (≤0.5–4), meropenem (≤0.0625–1), telithromycin (≤0.125–4), tetracycline (≤0.125–1; all susceptible [MICs in mg/L]). A resistant phenotype was recorded for trimethoprim/sulfamethoxazol (≥8/152) for all strains except *S*. *hongkongensis* DSM 26322^T^. Some strains of *S*. *moniliformis* displayed at least resistance or intermediate resistance to ciprofloxacin (2), erythromycin (16), gentamicin (4–8), nalidixic acid (32–128) and streptomycin (4–32).

#### Haemagglutination

Adhesive properties were detected in all 12 *S*. *moniliformis* strains tested. Erythrocytes of 11 different vertebrate species were agglutinated with varying intensity. The slide agglutination test represented results for spontaneous agglutination and most intense reactions could be observed in erythrocytes from turkeys, humans, guinea-pigs and pigs. Red blood cells from rats and chickens showed a strong reaction (++). C57BL/6J mice, known to represent a highly susceptible mouse strain towards streptobacillosis [18], were less strongly agglutinated compared to erythrocytes from the more resistant BALB/c mice (mostly + in contrast to mostly ++).

Results from the haemagglutination in microtiter plates differed in some way (Table 3). Again, erythrocytes from turkeys, humans and pigs, but also from rats and C57BL/6J mice proved to show the strongest haemagglutination reactions. Agglutination with erythrocytes from chickens, guinea-pigs and BALB/c mice was weaker compared to the results from slide agglutination tests.

**Table 3:** Adhesive properties of selected *S*. *moniliformis* strains from this study.

Erythrocytes from	DSM 12112^T^	NCTC 10773	NCTC 11194	CIP 81–99	NCTC 11941	ATCC 27747	IPDH 144/80	IPDH 109/83	ATCC 49567	CIP 55–48	ATCC 49940	Kun 3 (RIVM)
Turkey	++	++	++	++	++	++	++	++	++	++	++	++
turkey*	++	++	++	++	++	++	++	++	+	++	+	++
Human	++	+	++	++	++	++	++	++	+	++	+	++
human*	++	+	+	++	++	+	++	++	++	++	-	++
guinea pig	+	+	++	++	++	++	++	++	+	++	+	++
guinea pig*	++	+	++	++	++	++	++	++	++	++	+	++
mouse (C57BL/6)	++	+	++	++	++	++	++	++	++	++	+	+
mouse (C57BL/6)*	+	+	+	++	++	++	++	++	++	++	+	+
Rat	++	++	++	++	++	++	+	+	++	++	+	++
rat*	++	++	+	++	++	+	+	+	+	++	+	++
Pig	++	++	+	++	++	++	++	-	++	++	++	++
pig*	++	++	+	++	++	++	+	-	++	++	++	++
mouse (BALB/c)	+	+	+	++	++	+	++	++	++	++	-	+
mouse (BALB/c)*	+	+	+	++	+	+	++	++	++	++	+	+
Chicken	+	++	++	+	+	++	+	++	+	+	+	++
chicken*	+	++	++	+	+	++	++	++	+	++	+	++
Cattle	++	+	+	++	++	++	++	+	+	++	+	++
cattle*	++	+	+	++	+	++	+	+	+	++	+	++
Sheep	++	-	+	+	+	+	-	++	+	+	++	++
sheep*	+	+	+	+	-	+	+	+	+	+	+	+
Hamster	+	+	+	+	+	+	++	+	+	++	+	+
hamster*	+	+	+	+	+	+	++	+	+	+	-	+
Horse	-	-	+	+	-	+	+	+	+	+	-	+
horse*	+	-	+	+	-	+	+	+	-	+	-	+

Haemagglutination was tested in microtiter plates in the presence* and absence of 1% mannose; strong (++), moderate/weak (+) or no (-) reaction

In both experiments erythrocytes from cattle, sheep, hamsters and horses showed the weakest or even no agglutination. By adding mannose, a known agonist of a common adhesin receptor, no significant differences could be observed indicating mannose-resistant agglutination in all cases. No differences were observed between agglutination of erythrocytes from ‘original’ host species (from which respective strains were originally isolated) and other host red blood cells, but susceptibility was generally highest in species of potential hosts compared to non-host species.

#### MALDI-TOF MS

For MALDI-TOF MS, all 20 viable strains of *S*. *moniliformis* were identified to the species level with a score level between 2.0 and 2.4 using the direct smear and the extraction method for sample preparation. This was also true for strain AHL 370–1, which albeit clustered most distantly from all *S*. *moniliformis* strains. *Streptobacillus felis* 131000547^T^ as well as *S*. *hongkongensis* DSM 26322^T^ and *Sebaldella termitidis* ATCC 33386^T^ could not be identified yielding only score levels between 1.3 and 1.5 (database DB 5627, BrukerDaltonics). Following the manual inclusion of respective spectra of these strains to the database these were most closely related to *S*. *moniliformis*. A dendrogram including selected main spectra peaks (MSP) of the family *Leptotrichiaceae* from the Bruker database as well as from type strains of *S*. *felis*, *S*. *hongkongensis* and *Sebaldella termitidis* is depicted in Fig 1.

#### FT-IR

The comparison of the infrared-spectra of 22 viable strains of *Streptobacillus* spp. as well as the closely related *Sebaldella termitidis* ATCC 33386^T^ showed a clear separation into the three species *S*. *moniliformis*, *S*. *felis* and *S*. *hongkongensis* (Fig 2). Based on spectra within the genus *Streptobacillus* the spectral cloud derived from spectra of divergent strain AHL 370–1 could be delineated from all other strains (S1 Fig).

### Molecular characterization

#### Species specific PCR for *S*. *moniliformis*

Amplification of the specific target sequences resulted in characteristic amplicon sizes of approximately 269 and 1222 bp employing the PCR assays according to Kimura et al. [12] and Nicklas (cited in Rohde et al. [31]), respectively. All 23 *S*. *moniliformis* strains, the four divergent strains AHL 370–1, OGS16, KWG2 and KWG24 and also *S*. *felis* 131000547^T^ were found positive in both PCRs, whereas *S*. *hongkongensis* DSM 26322^T^ resulted in a specific amplicon in the PCR according to Nicklas but not to Kimura et al. (data not shown).

#### Phylogenetic analysis and determination of G/C contents

**Fig 3:**
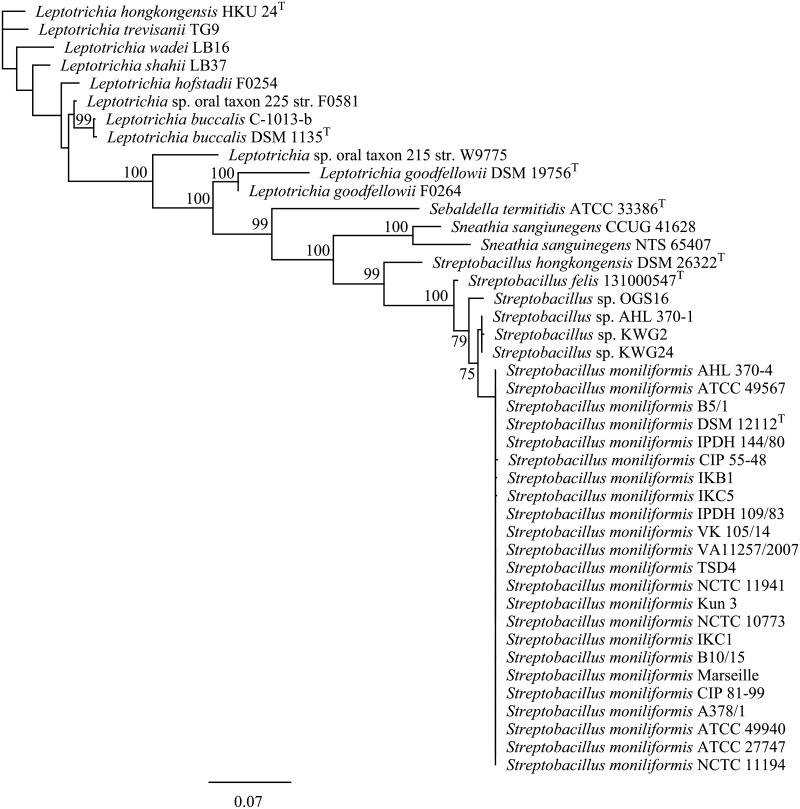
Maximum-likelihood tree showing the phylogenetic position within the family *Leptotrichiaceae*. The tree was generated in Geneious using PhyML [35] and based on 16S rRNA gene sequences. GenBank accession numbers are KR001904-1922, KP657489, KP657490-KP657495, and HG421076. Numbers at branch nodes refer to bootstrap values (100 replicates). Bar: 0.06 nucleotide substitutions per side.

Alignment of sequences of the partial 16S rRNA gene (1482 bp) revealed a sequence homology of 99.8–100% for 23 *S*. *moniliformis* strains under study (Fig 3). Three (AHL 370–1, KWG2, KWG24) and one (OGS16) divergent strains, respectively, were clustering separately (≥ 90% bootstrap support) and displayed sequence homology of 97.97–98.58% to the type strain DSM 12112^T^. The 16S rRNA gene homology between *S*. *moniliformis* DSM 12112^T^ compared to *S*. *felis* 131000547^T^ was 97.11%, whereas the homology between the *S*. *moniliformis* type strain and *S*. *hongkongensis* DSM 26322^T^ was 92.73%. The type strain of *S*. *hongkongensis* clustered closer with that of *Sneathia sanguinegens* than with *S*. *moniliformis* and *S*. *felis* 131000547^T^.

**Fig 4:**
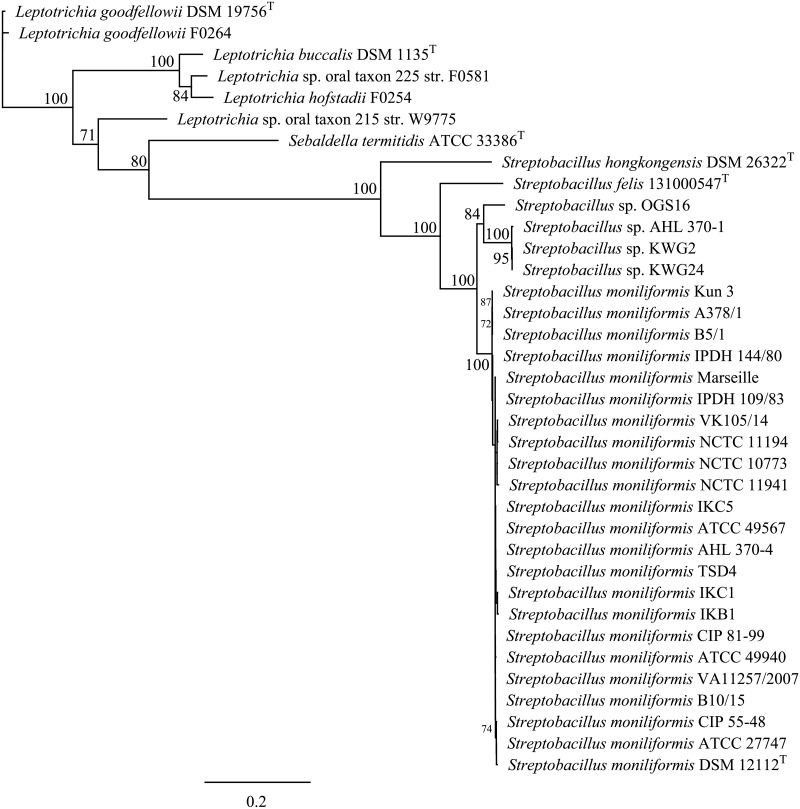
Phylogenetic trees based on concatenated sequences (4632 nt) of *groEL* (1.602 nt), *recA* (1.047 nt) and *gyrB* (1.983 nt), sequences including type strains of all species of the family *Leptotrichiaceae* showing the phylogenetic relationship of *Streptobacillus* species from this study. GenBank accession nos. are KR001923-1941 and KP657496-503 for *groEL*, KR001942-1960 and KP657504-511 for *recA*, KR001961-1979 and KP676101-108 for *gyrB* sequences. The tree was generated with the maximum-likelihood program PhyML (Substitution Model HKY85; number of bootstraps: 100) [35] after alignment of sequences with MAFFT v7.017, both implemented in Geneious. Numbers at nodes represent bootstrap values >70%. Bar: 0.2 substitutions per sequence position.

The outstanding position of the aforementioned four *S*. *moniliformis* strains as separate clusters was also supported by analysis of functional genes *groEL*, *gyrB*, and *recA*. The phylogenetic analysis based on concatenated sequences (4.632 bp) of these genes (*groEL* [1.602 bp], *recA* [1.047 bp], *gyrB* [1.983 bp]) (Fig 4) roughly revealed that strains AHL 370–1, KWG2 and KWG24 and also strain OGS16 clustered at two separate positions apart from the other members of the genus (100% bootstrap support), so that it was possible to clearly delineate *S*. *moniliformis*, divergent strain clusters 1 and 2, *S*. *hongkongensis* and *S*. *felis*.

G/C contents of *Streptobacillus* spp. obtained from non-published genomes from all strains revealed 25.7–28.9% (S2 Table), which is quite in accordance with previously determined values based on melting point analyses [26]. Average nucleotide identity (ANI) analysis revealed an overall DNA-DNA relatedness of 98.51 to 99.3% (recipr. 98.17–99.41%) between the type strain DSM 12112^T^ and all *S*. *moniliformis* strains under study, except for isolates AHL 370–1, KWG2, KWG24 and OGS16. ANI values below the cut-off for species boundary were calculated for *Streptobacillus* spp. isolates AHL 370–1, KWG2, KWG24 and OGS16 (88.21 to 89.1%; recipr. 87.89 to 89.87%). The average nucleotide identity between *S*. *moniliformis* type strain DSM 12112^T^ and *S*. *hongkongensis* DSM 26322^T^ and *S*. *felis* 131000547^T^ was 74.04% (recipr. 75.03%) and 82.0% (recipr. 82.02%), respectively (S2 Table).

#### Nucleotide sequence accession numbers

Partial 16S rRNA sequences of 27 *Streptobacillus* strains have been submitted to the GenBank database under accession nos. KR001904-KR001922, HG421076, KP657489, KP657491-KP657495, KT311784, CP001779. The *groEL*, *gyrB*, and *recA* sequences have been submitted under accession nos. KR001923-KR001941, KP657496-KP657497, KP657499-KP657503, KT311785, CP001779 (*groEL*), KR001942-KR001960, KP676101-KP676102, KP676104-KP676108, KT311786, CP001779 (*gyrB*), and KR001961-KR001979, KP657504-KP657505, KP657507-KP657511, KT311787, CP001779 (*recA*), respectively.

## Discussion

The aim of this study was a comparison of different strains of the genus *Streptobacillus* including representatives of *S*. *moniliformis* and two strains of the novel species *S*. *hongkongensis* and *S*. *felis*. Few studies have focused on strain diversity within the species *S*. *moniliformis*, the etiological agent of the worldwide occurring zoonosis RBF, and compared strains from different origins, also considering the occurrence of L-forms in this species [13–15, 26, 40, 41]. A similar broad spatiotemporal diversity of strains like in the present study never formed the basis for a comparison, also including novel members of the recently extended genus for the first time. Most previous studies revealed only little differences between strains with respect to their pheno- and genotype [15, 26]. Minor biochemical strain differences between studies were merely attributed to different preparations of culture media [12, 14, 15, 41–44] and might also be explained by difficulties due to the fastidious growth or the person reading the tests [26]. Even within the same study single physiological discrepancies were noted in repeated experiments with the same strain, thereby reflecting inconsistency in test results rather than discriminatory traits [26]. Protein profiles as observed by SDS-PAGE were found to be identical between *S*. *moniliformis* strains in a former study [13] and other chemotaxonomical investigations found a homologous fatty acid profile for all tested *S*. *moniliformis* strains of tetradecanoic acid (14:0), palmitic acid (16:0), octadecanoic acid with linoleic acid (18:2) and oleic acid (18:1), and stearic acid (18:0) [45–47].

However, comprehensive analyses of differences based on spectroscopic data of biomolecules as well as genotypic properties of strains were not yet published in international literature. The present study included 23 *S*. *moniliformis* strains from at least five different host species that cover isolations over the past 90 years from almost all subcontinents as well as the type strains of *S*. *hongkongensis* and *S*. *felis* and four divergent strains that presumably belong to two yet undescribed species. Classical biochemistry of most key reactions was in accordance with published results, although we also observed significant growth differences depending on the kind of carbohydrate preparation (data not shown). Therefore, standardized test systems for physiological typing of *S*. *moniliformis* were included. Earlier studies involved API 20E for assessing biochemical profiles [12]. As this test requires viable bacteria and as previously chosen culture conditions were not appropriate for the growth of *S*. *moniliformis*, published results of this method may be interpreted with caution. We have successfully employed commercially available systems API ZYM [6, 12, 14, 20] and VITEK2-compact (NHI profile) [9] and basically validated the Micronaut system for a novel application with *Streptobacillus*. All these systems use biochemical end-point measurements and are thus independent from bacterial growth though standardized and the Micronaut system earlier proved to be suitable for bacterial species and biotype discrimination [48]. The designed identification panel of this system could unequivocally discriminate members of the genus *Streptobacillus* from other bacterial species by considering growth characteristics (fastidious capnophilic growth, occurrence of L-forms, “puff-ball”- or “bread crumb”-like appearance in liquid media [2, 3, 12, 14, 15] and eleven biochemical reactions (Table 2). Briefly, five of these reactions were identical among all *S*. *moniliformis* as well as *S*. *felis* 131000547^T^. However, most other physiological tests showed too diverse reactions to discriminate species or biotypes, especially because the variability of *S*. *hongkongensis*, *S*. *felis* and the yet undescribed members could not or not sufficiently be evaluated by one or three strains each. Like in other studies, we could confirm major uniformity between strains of *S*. *moniliformis* for most physiological reactions. Nevertheless, minor discrepancies were observed, some of which were earlier reported [12, 14, 42–44] and can–in the case of API ZYM reactions–be explained by differences in semi quantitative reading of results, too [12, 14, 15, 41–43, 49].

Antimicrobial resistance profiles revealed that *S*. *moniliformis* is susceptible to all β-lactam antibiotics [14], and no β-lactamase activity could be demonstrated so far [50]. Penicillin G was repeatedly reported as the most active antimicrobial substance in *in vitro* tests, which further supports its use as the drug of choice in the treatment of RBF and HF, followed by tetracycline [2]. The majority of studies have performed antimicrobial susceptibility testing (AST) with rather old-fashioned methods without determining MIC values and not including all relevant actual agents and novel *Streptobacillus* species. In order to provide a more up-to-date picture we included recently isolated strains and carried out AST by broth microdilution with a commercially available test. The strains from this study were very similar in their resistance pattern and largely confirmed a generally good therapeutic basis. However, at least some isolates were resistant or intermediate resistant to ciprofloxacin, erythromycin, gentamicin, nalidixic acid and streptomycin. Interestingly and in contrast to Woo et al. [9], *S*. *hongkongensis* DSM 26322^T^ turned out to be the only member of the genus under study that was trimethoprim/sulfamethoxazol-sensitive. Based on chemoresistance, biochemical and chemotaxonomic results from this and former studies other traits should be propagated, both for diagnostics as well as for strain comparisons in *Streptobacillus*. One of these applicable methods is the acquisition of MALDI-TOF and FT-IR spectra that were initially included on a large scale basis. By MALDI-TOF MS all tested strains of *S*. *moniliformis* could be assigned with high accuracy to species level. This was also true for representative spectra of *S*. *hongkongensis* and *S*. *felis* [10] and facilitated also discrimination of one of the undescribed *Streptobacillus* sp. (strain AHL 370–1). In contrast to MALDI-TOF MS, where the spectral information mirrors protein components, spectra generated by FT-IR include information from a broad variety of main component biomolecules [51]. Again, spectra from type strains of *S*. *hongkongensis* and *S*. *felis* were shown to be distinct, but closely adjacent to those from all other *S*. *moniliformis* strains from this study, whereas spectra from strain AHL 370–1 could be noted as a small outgroup nearby the *S*. *moniliformis* cluster (Fig 2). It remains to be elucidated, whether strains KWG2, KWG24 and OGS16, from which only DNA was available, will confirm spectral traits found here. Interestingly, despite displaying a unique profile in electrophoretic protein patterns [13] no antigenic differences could be observed for strain AHL 370–1 [52].

Based on highly uniform electrophoretic protein patterns it was hypothesized that *S*. *moniliformis* strains could be grouped with respect to host species, geographic origin, disease pattern and route of infection, i.e. isolates from cases of RBF and HF should be clearly assigned by protein profiling [13]. We and others have not found evidence for these assumptions [15, 26, 52]. On the other hand, especially differences of HF- versus RBF-strains are unlikely because rats represent the source of infection in both cases and disparities could better be explained by different gene expression following oral or parenteral infection [3] or simply by too few HF-strains under study. Additionally, the time between infection and strain isolation from the host following rat exposure is usually too short to facilitate adaptation of strains and expression of a different phenotype. A number of studies have, nevertheless, proven that strains isolated from susceptible host species were able to cause infection in rodents, thereby partially fulfilling Koch’s postulates [16, 18]. As hypothesized by Nolan et al. [50], no phylotypes of *S*. *moniliformis* were yet detected indicating that this species is rarely found in the environment outside of its natural hosts.

A number of studies have used 16S rRNA gene sequencing as a diagnostic tool for species determination of *S*. *moniliformis* in strains and clinical specimens [53–56]. Both PCR protocols targeting the 16S rRNA gene published as specific for *S*. *moniliformis* were suitable to detect all strains from humans, rats, mice, a spinifex hopping mouse and turkeys from this study and also *S*. *felis* [10]. In contrast, *S*. *hongkongensis* was only detected by the PCR described by Rohde et al. [31] suggesting that both PCR are merely genus rather than species-specific. In order to elucidate the sufficiency, usefulness and discriminatory power of marker genes for inter- as well as intraspecific analysis within the genus *Streptobacillus*, sequence data from all strains were analysed. Within the phylum Fusobacteria sequencing of 16S rDNA, 16S-23S rDNA internal transcribed spacer, *gyrB*, *groEL*, *recA*, *rpoB*, conserved indels and genes for group-specific proteins, 43-kDa outer membrane protein and zinc protease have been proposed for species identification or phylogenetic analysis [57–65] and eleven whole genome sequences were generated to date. We have used functional genes 16S rRNA, *groEL*, *recA* and *gyrB*, which unequivocally could discriminate the strains from this study to species level. Fueled by the work presented here and further studies we have identified two other candidate species of *Streptobacillus*, one of which will be described shortly, thereby significantly extending the knowledge of this former monotypic genus. A more detailed insight into some of these candidates was solely possible by comparison of further genes, which otherwise would have been missed solely by 16S rRNA gene sequencing. Interestingly, a previous study came to the conclusion that disparities in 16S rRNA might in fact be based on different co-evolution of strains in different rat host species as suggested by Kimura et al. (2008). Indeed were isolates directly or indirectly (mouse after rat bite) obtained from brown rats (*Rattus norvegicus*; strains no. 14, 16, 18, 19, 21–24) belonging to *S*. *moniliformis*, but for the remaining *S*. *moniliformis* strains the exact source rat species remained obscure. Variations of the 16S rRNA gene were mainly observed in the region at nucleotide positions 1 to 300 [12], which could also be confirmed in our study and involved four strains (AHL 370–1, KWG2, KWG24, OGS16 cf. Fig 3). Their outstanding position from *S*. *moniliformis* was also supported by analysis of other functional genes (cf. Fig 4), but these four strains represented in fact two separate clusters. Interestingly, we found that strain AHL 370–1 from the spinifex hopping mouse clustered identical with strains KWG2 and KWG24 and respective genomes were also indistinguishable as obtained by ANI.

We therefore believe that strains OGS16, KWG2 and KWG24 are not exclusively restricted to *R*. *rattus*. This assumption is, on the other hand, also supported by the complete absence of human isolates resembling the 16S rRNA pattern of these three strains despite the rarity of *R*. *rattus* in many regions worldwide. Further epidemiologically based studies are required to elucidate the true prevalence of these microorganisms in different rat as well as further rodent host species. However, at present there is indeed no evidence that these genetic differences are geographically related, because the yet undescribed species were found in close proximity in Japan [12] and strain AHL 370–1 from this study originated from Australia.

With respect to G/C contents our investigations revealed a nearly identical G/C content of 25.7–28.9 mol% in all investigated *Streptobacillus* strains. The G/C content of *Sebaldella termitidis* was only 33.38 mol% [26, 66], thereby deceeding the classical value for species discrimination of 10 mol% [67]. Results from the literature of *S*. *hongkongensis* revealed G/C content of 26.0 [9, 10] and *S*. *felis* had 26.4 mol%. Analogous results could also be confirmed by ANI. A value of 70% DDH was proposed by Wayne et al. (1987) [68] as a recommended standard for delineating species. Goris et al. (2007) [37] could demonstrate a close relationship between DDH values and ANI in that the recommended cut-off point of 70% for species discrimination corresponded to 95% ANI. The 95–96% species boundary is also supported by Richter & Rosselló-Móra [38], who have developed an alignment free interface to calculate ANI also used in this study, which should even work for taxonomic issues on a 20% partial genome of strains under study. In contrast to the highly homologous group of *S*. *moniliformis* strains not only *S*. *hongkongensis* and *S*. *felis* but again also AHL 370–1, KWG2, KWG24 and OGS16 had significantly lower ANI values thereby clearly pointing towards separate species apart from *S*. *moniliformis*.

Concerning pathogenicity one might suspect haemolysis as a possible marker of virulence [3]. Indeed were haemolytic strains of *S*. *moniliformis*, *S*. *hongkongensis* and *S*. *felis* involved in clinical disease in a rat (strain no. 14, ATCC49940) [15], a dog [7], a cat [39] and a human [10], but other clinical isolates, especially those causing severe or even fatal disease turned out to be non-haemolytic so that other virulence factors apparently play a more important role. These might include the extraordinary high amount of DNase in all strains, which was released even independently of bacterial growth [26]. However, another possible relationship was detected during haemagglutination experiments that could also be explained as trait of virulence: susceptibility of erythrocytes to haemagglutination was generally highest in species serving as potential hosts like humans, mice, rats, turkeys and guinea-pigs compared to non-host species suggesting a predisposing role for potential host species. No significant differences were observed between agglutination of erythrocytes from ‘original’ host species, i.e. from which respective strains were originally isolated and other host red blood cells, which again gives rise to the assumption that infectious strains do at least not rapidly adapt to their hosts following infection. However, these experiments unequivocally suggest the presence of adhesins, a mechanism involved in pathogenicity which is a prerequisite for the ‘successful’ infection of a host. Indeed, there appear to remain other factors besides adhesins as can be concluded from infections in hosts with missing host specificity. These might include not yet identified genetic factors at the host side which can be concluded from differences in susceptibility to infection like for instance the genetically diverse, highly susceptible C57BL/6J mice compared to BALB/c mice [18, 69].

## Conclusion

Rat bite fever represents a significant public health threat that is under-diagnosed in humans and animal species. We have analyzed the largest-ever collection of strains with respect to time and geography of this rarely isolated microorganism. In a polyphasic approach we could show that some recently described as well as novel candidate species besides *S*. *moniliformis* could be differentiated, which are not restricted to 16S rRNA gene differences. It will be challenging to note the true prevalence of these novel members in human and animal infections. Systematical data for several of the strains from this study with respect to G/C contents as well as haemagglutination were assessed before [26], but never made available to the public at large, which will hopefully help in future research to further elucidate virulence properties of these neglected bacterial species. Growth characteristics together with traditional physiological methods and also standardized biochemistry are suitable for genus determination, but do not possess enough discriminatory power to sufficiently differentiate *Streptobacillus* spp. We have shown that the strains of *S*. *moniliformis* as well as putative and designated type strains of the novel species could be easily classified with modern genotypic and phenotypic methods including sequence analysis of selected functional genes as well as FT-IR and MALDI-TOF MS. The latter proved to have the potential to easily diagnose the novel species *S*. *hongkongensis* and *S*. *felis* and also at least one yet undescribed species. Further genetic studies on *Streptobacillus* should include investigations on possible virulence genes and differences in pathogenic mechanism between strains.

## Supporting Information

S1 Fig Linear discriminant analysis (LDA) of 182 infrared spectra of 22 *Streptobacillus* isolates. The wave numbers 550–1800 cm^-1^ and 2800–3200 cm^-1^ of second derivative spectra were selected and vector normalized. After a principal component analysis, the first 40 components were used for the LDA. In this LDA every isolate was defined as one group. Spectra of *Streptobacillus felis* are represented by diamonds, *Streptobacillus hongkongenis* by triangles, *Streptobacillus* sp. (AHL 370–1) by circles and *S*. *moniliformis* by dots. Note that LDA axis 1 and 3 are shown, because axis 3 contains the differences between *Streptobacillus* sp. (AHL 370–1) and *S*. *moniliformis*.
(TIF)

S1 Table Antimicrobial susceptibility testing of *Streptobacillus moniliformis* strains and of reference strains *Streptobacillus felis* 131000547^T^, *Streptobacillus hongkongensis* DSM 26322^T^ and *Sebaldella termitidis* NCTC 11300^T^. Broth microdilution susceptibility testing was performed with the Merlin Micronaut system; results were interpreted according to Clinical and Laboratory Standards Institute (CLSI) MIC criteria based on CLSI MIC interpretive standards for other non-*Enterobacteriaceae* and anaerobes [25]. AZM: azithromycin, CIP: ciprofloxacin, CLI: clindamycin, CMP: chloramphenicol, ERY: erythromycin, GEN: gentamicin, MER: meropenem, NAL: nalidixic acid, STR: streptomycin, T/S: trimethoprim/sulfamethoxazole, TEL: telithromycin, TET: tetracycline, R: resistant, I: intermediate susceptible, S: susceptible phenotype, MIC values in mg/L.
(DOCX)

S2 Table Guanin/cytosin (G/C) contents of *Streptobacillus* spp. isolates and average nucleotide identity (ANI) of all strains under study to *S*. *moniliformis* type strain DSM 12112^T^. (DOCX)

## References

[1] Levaditi C, Nicolau S, Poincloux P. Sur le rôle étiologique de *Streptobacillus moniliformis* (nov. spec.) dans l'érythème polymorphe aigu septicémique. C R Acad Sci. 1925;180:1188–90.

[2] Elliott SP. Rat bite fever and *Streptobacillus moniliformis* . Clin Microbiol Rev. 2007;20(1):13–22. Epub 2007/01/16. 10.1128/cmr.00016-06 17223620 PMC1797630

[3] Gaastra W, Boot R, Ho HT, Lipman LJ. Rat bite fever. Vet Microbiol. 2009;133(3):211–28. Epub 2008/11/15. 10.1016/j.vetmic.2008.09.079 . 19008054

[4] Torres L, Lopez AI, Escobar S, Marne C, Marco ML, Perez M, et al Bacteremia by *Streptobacillus moniliformis*: first case described in Spain. Eur J Clin Microbiol Infect Dis. 2003;22(4):258–60. Epub 2003/04/24. . 12709841 10.1007/s10096-003-0891-9

[5] Bleich A, Nicklas W. Zoonoses transmitted by mouse and rat maintained as laboratory or pet animals [in German]. Berl Münch Tierärztl Wochenschr. 2008;121(7–8):241–55. Epub 2008/08/21. . 18712260

[6] Hayashimoto N, Yoshida H, Goto K, Takakura A. Isolation of *Streptobacillus moniliformis* from a pet rat. J Vet Med Sci. 2008;70(5):493–5. Epub 2008/06/06. . 18525173 10.1292/jvms.70.493

[7] Ditchfield J, Lord LH, McKay KA. *Streptobacillus moniliformis* infection in a dog. Can Vet J. 1961;2(12):457–9. Epub 1961/12/01. 17421433 PMC1585851

[8] Washburn RG. *Streptobacillus moniliformis* (rat-bite fever) In: Mandell GL, Bennett JE, Dolin RG, editors. Principles and practice of infectious diseases Vol 2 New York: Churchill Livingstone; 1995 p. 2084–6.

[9] Woo PC, Wu AK, Tsang CC, Leung KW, Ngan AH, Curreem SO, et al *Streptobacillus hongkongensis* sp. nov., isolated from patients with quinsy and septic arthritis in Hong Kong, and emended descriptions of the genus *Streptobacillus* and the species *Streptobacillus moniliformis* . Int J Syst Evol Microbiol. 2014 10.1099/ijs.0.061242-0 . 24912824

[10] Eisenberg T, Glaeser S, Nicklas W, Mauder N, Contzen M, Aledelbi K, et al *Streptobacillus felis* sp. nov. isolated from a cat with pneumonia. Int J Syst Evol Microbiol—accepted manuscript. 2015. 10.1099/ijs.0.000238 25858245

[11] Dewhirst FE, Klein EA, Thompson EC, Blanton JM, Chen T, Milella L, et al The canine oral microbiome. PLoS One. 2012;7(4):e36067 Epub 2012/05/05. 10.1371/journal.pone.0036067 PONE-D-12-02767 [pii]. 22558330 PMC3338629

[12] Kimura M, Tanikawa T, Suzuki M, Koizumi N, Kamiyama T, Imaoka K, et al Detection of *Streptobacillus* spp. in feral rats by specific polymerase chain reaction. Microbiol Immunol. 2008;52(1):9–15. Epub 2008/03/21. 10.1111/j.1348-0421.2008.00005.x . 18352907

[13] Costas M, Owen RJ. Numerical analysis of electrophoretic protein patterns of *Streptobacillus moniliformis* strains from human, murine and avian infections. J Med Microbiol. 1987;23(4):303–11. Epub 1987/06/01. . 3585963 10.1099/00222615-23-4-303

[14] Edwards R, Finch RG. Characterisation and antibiotic susceptibilities of *Streptobacillus moniliformis* . J Med Microbiol. 1986;21(1):39–42. Epub 1986/02/01. . 3950962 10.1099/00222615-21-1-39

[15] Wullenweber M. *Streptobacillus moniliformis*—a zoonotic pathogen. Taxonomic considerations, host species, diagnosis, therapy, geographical distribution. Lab Animal. 1995;29(1):1–15. Epub 1995/01/01. . 7707673 10.1258/002367795780740375

[16] Yamamoto R, Clark GT. *Streptobacillus moniliformis* infection in turkeys. Vet Rec. 1966;79(4):95–100. Epub 1966/07/23. . 5968147 10.1136/vr.79.4.95

[17] Hopkinson WI, Lloyd JM. *Streptobacillus moniliformis* septicaemia in spinifex hopping mice (*Notomys alexis*). Aust Vet J. 1981;57(11):533–4. Epub 1981/11/01. . 7342940 10.1111/j.1751-0813.1981.tb05802.x

[18] Wullenweber M, Kaspareit-Rittinghausen J, Farouq M. *Streptobacillus moniliformis* epizootic in barrier-maintained C57BL/6J mice and susceptibility to infection of different strains of mice. Lab Anim Sci. 1990;40(6):608–12. Epub 1990/11/01. . 2172624

[19] Boot R, Oosterhuis A, Thuis HC. PCR for the detection of *Streptobacillus moniliformis* . Lab Anim. 2002;36(2):200–8. Epub 2002/04/12. . 11943086 10.1258/0023677021912352

[20] Wullenweber M, Jonas C, Kunstyr I. *Streptobacillus moniliformis* isolated from otitis media of conventionally kept laboratory rats. J Exp Anim Sci. 1992;35(1):49–57. Epub 1992/03/01. . 1534999

[21] Kondruweit M, Weyand M, Mahmoud FO, Geissdorfer W, Schoerner C, Ropers D, et al Fulminant endocarditis caused by *Streptobacillus moniliformis* in a young man. J Thorac Cardiovasc Surg. 2007;134(6):1579–80. Epub 2007/11/21. 10.1016/j.jtcvs.2007.08.010 . 18023687

[22] Loridant S, Jaffar-Bandjee MC, La Scola B. Shell vial cell culture as a tool for *Streptobacillus moniliformis* "resuscitation". Am J Trop Med Hyg. 2011;84(2):306–7. Epub 2011/02/05. 10.4269/ajtmh.2011.10-0466 21292904 PMC3029187

[23] Manafi M, Kneifel W, Bascomb S. Fluorogenic and chromogenic substrates used in bacterial diagnostics. Microbiological reviews. 1991;55(3):335–48. 1943991 10.1128/mr.55.3.335-348.1991 PMC372823

[24] Wellinghausen N, Pietzcker T, Poppert S, Belak S, Fieser N, Bartel M, et al Evaluation of the Merlin MICRONAUT system for rapid direct susceptibility testing of gram-positive cocci and gram-negative bacilli from positive blood cultures. J Clin Microbiol. 2007;45(3):789–95. 10.1128/JCM.01856-06 17202283 PMC1829091

[25] Anonymous. Performance standards for antimicrobial susceptibility testing; 23rd informational supplement. CLSI document 2013;M100–S23.

[26] Hofmann N. Phenotypical and molecular taxonomic investigations on the systematic status of *Streptobacillus moniliformis*, the agent of rat-bite-fever [in German]. Thesis Dr. rer. nat., Faculty of Biology, Leibniz Universität Hannover 1994.

[27] Kuhm AE, Suter D, Felleisen R, Rau J. Identification of *Yersinia enterocolitica* at the species and subspecies levels by Fourier transform infrared spectroscopy. Appl Environ Microbiol. 2009;75(18):5809–13. 10.1128/AEM.00206-09 19617388 PMC2747871

[28] Helm D, Labischinski H, Schallehn G, Naumann D. Classification and identification of bacteria by Fourier-transform infrared spectroscopy. J Gen Microbiol. 1991;137(1):69–79. . 1710644 10.1099/00221287-137-1-69

[29] Rau J, Perz R, Klittich G, Contzen M. Cereulide forming presumptive *Bacillus cereus* strains from food—differentiating analyses using cultural methods, LC-MS/MS, PCR, and infrared spectroscopy in consideration of thermotolerant isolates [in German]. Berl Münch Tierärztl Wochenschr. 2009;122(1–2):25–36. . 19226933

[30] Ward JH. Hierarchical grouping to optimize an objective function. J Amer Statist Assoc 1963;58:236–44.

[31] Rohde J, Rapsch C, Fehr M. Case report: Abscessation due to *Streptobacillus moniliformis* in a rat [in German]. Prakt Tierarzt. 2008;89(6):466–73.

[32] Aziz RK, Bartels D, Best AA, DeJongh M, Disz T, Edwards RA, et al The RAST Server: rapid annotations using subsystems technology. BMC Genomics. 2008;9:75 10.1186/1471-2164-9-75 18261238 PMC2265698

[33] Kearse M, Moir R, Wilson A, Stones-Havas S, Cheung M, Sturrock S, et al Geneious Basic: an integrated and extendable desktop software platform for the organization and analysis of sequence data. Bioinformatics. 2012;28(12):1647–9. 10.1093/bioinformatics/bts199 22543367 PMC3371832

[34] Katoh K, Misawa K, Kuma K, Miyata T. MAFFT: a novel method for rapid multiple sequence alignment based on fast Fourier transform. Nucleic Acids Res. 2002;30(14):3059–66. 12136088 10.1093/nar/gkf436 PMC135756

[35] Guindon S, Gascuel O. A simple, fast, and accurate algorithm to estimate large phylogenies by maximum likelihood. Systematic biology. 2003;52(5):696–704. . 14530136 10.1080/10635150390235520

[36] Hasegawa M, Kishino H, Yano T. Dating of the human-ape splitting by a molecular clock of mitochondrial DNA. Journal of molecular evolution. 1985;22(2):160–74. . 3934395 10.1007/BF02101694

[37] Goris J, Konstantinidis KT, Klappenbach JA, Coenye T, Vandamme P, Tiedje JM. DNA-DNA hybridization values and their relationship to whole-genome sequence similarities. Int J Syst Evol Microbiol. 2007;57(Pt 1):81–91. 10.1099/ijs.0.64483-0 . 17220447

[38] Richter M, Rossello-Mora R. Shifting the genomic gold standard for the prokaryotic species definition. Proc Natl Acad Sci U S A. 2009;106(45):19126–31. 10.1073/pnas.0906412106 19855009 PMC2776425

[39] Eisenberg T, Nesseler A, Nicklas W, Spamer V, Seeger H, Zschöck M. *Streptobacillus* sp. isolated from a cat with pneumonia. J Clin Microbiol Case Reports. 2014;2014:1–7. 10.1099/jmmcr.0.000562

[40] Roughgarden JW. Antimicrobial therapy of ratbite fever. A review. Archives of Internal Medicine. 1965;(116):39–54. 14338952 10.1001/archinte.1965.03870010041007

[41] Cohen RL, Wittler RG, Faber JE. Modified biochemical tests for characterization of L-phase variants of bacteria. Appl Microbiol. 1968;16(11):1655–62. Epub 1968/11/01. 4302280 10.1128/am.16.11.1655-1662.1968 PMC547735

[42] Lambe DW Jr, McPhedran AM, Mertz JA, Stewart P. *Streptobacillus moniliformis* isolated from a case of Haverhill fever: biochemical characterization and inhibitory effect of sodium polyanethol sulfonate. Am J Clin Pathol. 1973;60(6):854–60. Epub 1973/12/01. . 4586017 10.1093/ajcp/60.6.854

[43] Smith CD, Sampson CC. Studies of *Streptobacillus moniliformis* from a case of human rat-bite fever. Am J Med Technol. 1960;26:47–50. Epub 1960/01/01. . 13831909

[44] Wittler RG, Cary SG. Genus *Streptobacillus* Levaditi In: Buchanan RE, Gibbons NE, editors. Bergey's manual of determinative bacteriology, 8th edition Baltimore: Williams & Wilkins Co; 1974 p. 378–81.

[45] Pins MR, Holden JM, Yang JM, Madoff S, Ferraro MJ. Isolation of presumptive *Streptobacillus moniliformis* from abscesses associated with the female genital tract. Clin Infect Dis. 1996;22(3):471–6. Epub 1996/03/01. . 8852965 10.1093/clinids/22.3.471

[46] Rowbotham TJ. Rapid identification of *Streptobacillus moniliformis* . Lancet. 1983;2(8349):567 Epub 1983/09/03. . 6136713 10.1016/s0140-6736(83)90591-3

[47] Rygg M, Bruun CF. Rat bite fever (*Streptobacillus moniliformis*) with septicemia in a child. Scand J Infect Dis. 1992;24(4):535–40. Epub 1992/01/01. . 1411321 10.3109/00365549209052641

[48] Al Dahouk S, Scholz HC, Tomaso H, Bahn P, Gollner C, Karges W, et al Differential phenotyping of *Brucella* species using a newly developed semi-automated metabolic system. BMC Microbiol. 2010;10:269 Epub 2010/10/26. 10.1186/1471-2180-10-269 1471-2180-10-269 [pii]. 20969797 PMC2984481

[49] Greenwood JR, Harvey SM. *Streptobacillus moniliformis* In: Dworkin M, Falkow M, Rosenberg SE, Schleifer KH, Stackebrandt E, editors. The Prokaryotes, a handbook on the biology of bacteria. volume 7, Proteobacteria. New York, NY: Springer; 2006 p. 983–5.

[50] Nolan M, Gronow S, Lapidus A, Ivanova N, Copeland A, Lucas S, et al Complete genome sequence of *Streptobacillus moniliformis* type strain (9901). Stand Genomic Sci. 2009;1(3):300–7. Epub 2009/01/01. 10.4056/sigs.48727 21304670 PMC3035246

[51] Naumann D. Infrared spectroscopy in microbiology In: Meyers RA, editor. Encyclopedia of Analytical Chemistry. Chichester: John Wiley & Sons Ltd; 2000 p. 102–31.

[52] Boot R, Bakker RH, Thuis H, Veenema JL, De Hoog H. An enzyme-linked immunosorbent assay (ELISA) for monitoring rodent colonies for *Streptobacillus moniliformis* antibodies. Lab Anim. 1993;27(4):350–7. Epub 1993/10/01. . 8277708 10.1258/002367793780745516

[53] Berger C, Altwegg M, Meyer A, Nadal D. Broad range polymerase chain reaction for diagnosis of rat-bite fever caused by *Streptobacillus moniliformis* . Pediatr Infect Dis J. 2001;20(12):1181–2. Epub 2001/12/12. . 11740332 10.1097/00006454-200112000-00021

[54] Glasman PJ, Thuraisingam A. Rat bite fever: a misnomer? BMJ Case Rep. 2009;2009 Epub 2009/01/01. 10.1136/bcr.04.2009.1795 22180758 PMC3029161

[55] Nakagomi D, Deguchi N, Yagasaki A, Harada K, Shibagaki N, Kimura M, et al Rat-bite fever identified by polymerase chain reaction detection of *Streptobacillus moniliformis* DNA. J Dermatol. 2008;35(10):667–70. Epub 2008/11/20. 10.1111/j.1346-8138.2008.00541.x . 19017047

[56] Wallet F, Savage C, Loiez C, Renaux E, Pischedda P, Courcol RJ. Molecular diagnosis of arthritis due to *Streptobacillus moniliformis* . Diagn Microbiol Infect Dis. 2003;47(4):623–4. Epub 2004/01/09. . 14711486 10.1016/s0732-8893(03)00167-6

[57] Woo PC, Wong SS, Teng JL, Leung KW, Ngan AH, Zhao DQ, et al *Leptotrichia hongkongensis* sp. nov., a novel *Leptotrichia* species with the oral cavity as its natural reservoir. J Zhejiang Univ Sci B. 2010;11(6):391–401. Epub 2010/05/28. 10.1631/jzus.B1000056 20506569 PMC2880351

[58] Conrads G, Claros MC, Citron DM, Tyrrell KL, Merriam V, Goldstein EJ. 16S-23S rDNA internal transcribed spacer sequences for analysis of the phylogenetic relationships among species of the genus *Fusobacterium* . Int J Syst Evol Microbiol. 2002;52(Pt 2):493–9. Epub 2002/04/05. . 11931161 10.1099/00207713-52-2-493

[59] Sun D, Zhang H, Lv S, Wang H, Guo D. Identification of a 43-kDa outer membrane protein of *Fusobacterium necrophorum* that exhibits similarity with pore-forming proteins of other *Fusobacterium* species. Res Vet Sci. 2013;95(1):27–33. Epub 2013/02/26. 10.1016/j.rvsc.2013.01.016 . 23433684

[60] Kim HS, Lee DS, Chang YH, Kim MJ, Koh S, Kim J, et al Application of *rpoB* and zinc protease gene for use in molecular discrimination of *Fusobacterium nucleatum* subspecies. J Clin Microbiol. 2010;48(2):545–53. Epub 2009/12/04. 10.1128/jcm.01631-09 19955278 PMC2815611

[61] Shah HN, Olsen I, Bernard K, Finegold SM, Gharbia S, Gupta RS. Approaches to the study of the systematics of anaerobic, gram-negative, non-sporeforming rods: current status and perspectives. Anaerobe. 2009;15(5):179–94. Epub 2009/08/22. 10.1016/j.anaerobe.2009.08.003 . 19695337

[62] Strauss J, White A, Ambrose C, McDonald J, Allen-Vercoe E. Phenotypic and genotypic analyses of clinical *Fusobacterium nucleatum* and *Fusobacterium periodonticum* isolates from the human gut. Anaerobe. 2008;14(6):301–9. Epub 2008/12/31. 10.1016/j.anaerobe.2008.12.003 . 19114111

[63] Jin J, Haga T, Shinjo T, Goto Y. Phylogenetic analysis of *Fusobacterium necrophorum*, *Fusobacterium varium* and *Fusobacterium nucleatum* based on *gyrB* gene sequences. J Vet Med Sci. 2004;66(10):1243–5. Epub 2004/11/06. . 15528856 10.1292/jvms.66.1243

[64] Jalava J, Eerola E. Phylogenetic analysis of *Fusobacterium alocis* and *Fusobacterium sulci* based on 16S rRNA gene sequences: proposal of *Filifactor alocis* (Cato, Moore and Moore) comb. nov. and *Eubacterium sulci* (Cato, Moore and Moore) comb. nov. Int J Syst Bacteriol. 1999;49 Pt 4:1375–9. Epub 1999/11/11. . 10555315 10.1099/00207713-49-4-1375

[65] Lawson PA, Gharbia SE, Shah HN, Clark DR, Collins MD. Intrageneric relationships of members of the genus *Fusobacterium* as determined by reverse transcriptase sequencing of small-subunit rRNA. Int J Syst Bacteriol. 1991;41(3):347–54. Epub 1991/07/01. . 1715737 10.1099/00207713-41-3-347

[66] Harmon-Smith M, Celia L, Chertkov O, Lapidus A, Copeland A, Glavina Del Rio T, et al Complete genome sequence of *Sebaldella termitidis* type strain (NCTC 11300). Stand Genomic Sci. 2010;2(2):220–7. Epub 2011/02/10. 10.4056/sigs.811799 ; PubMed Central PMCID: PMCPmc3035275. 21304705 PMC3035275

[67] Schleifer KH, Stackebrandt E. Molecular systematics of prokaryotes. Annual review of microbiology. 1983;37:143–87. 10.1146/annurev.mi.37.100183.001043 . 6416143

[68] Wayne LG, Brenner DJ, Colwell RR, Grimont PAD, Kandler O, Krichevsky MI, et al Report of the ad hoc committee on reconciliation of approaches to bacterial systematics. Int J Syst Bacteriol 1987;37:463–4.

[69] Wullenweber M, Hedrich HJ, Reetz IC. Susceptibility to streptobacillosis of mice is highly influenced by genetic factors. AALAS Bulletin. 1991;30:43.

