# Supplementary figures and images for: Phenotypic and Genotypic Characteristics of Members of the Genus Streptobacillus
Source: PLoS One. 2015 Aug 7;10(8):e0134312. doi: 10.1371/journal.pone.0134312 (PMC4529157; doi:10.1371/journal.pone.0134312)

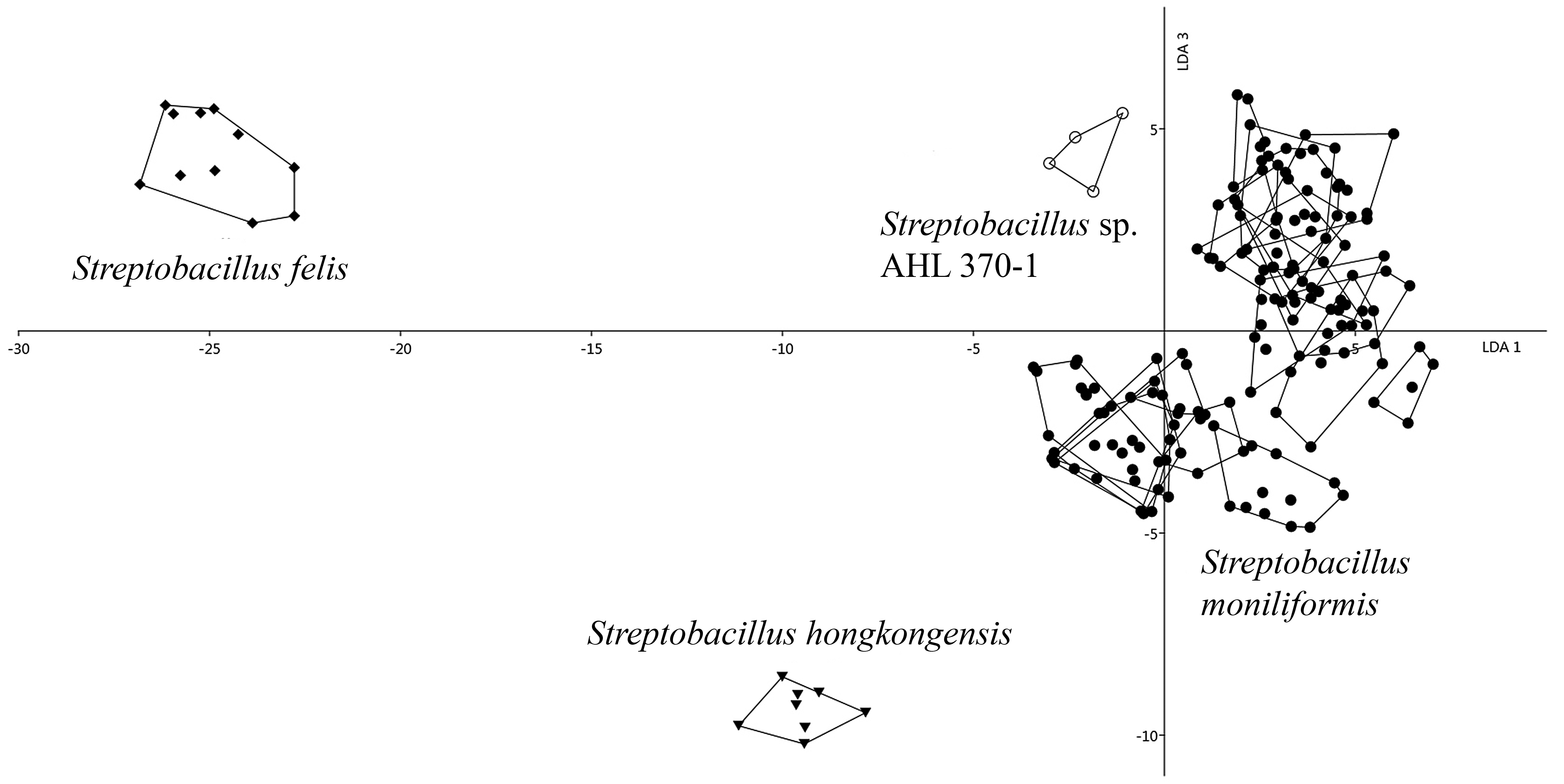

Supplement: S1 Fig — The wave numbers 550–1800 cm-1 and 2800–3200 cm-1 of second derivative spectra were selected and vector normalized. After a principal component analysis, the first 40 components were used for the LDA. In this LDA every isolate was defined as one group. Spectra of Streptobacillus felis are represented by diamonds, Streptobacillus hongkongenis by triangles, Streptobacillus sp. (AHL 370–1) by circles and S. moniliformis by dots. Note that LDA axis 1 and 3 are shown, because axis 3 contains the differences between Streptobacillus sp. (AHL 370–1) and S. moniliformis. (TIF) [file pone.0134312.s001.tif]
