# Supplementary material for: Phenotypic and Genotypic Characteristics of Members of the Genus Streptobacillus
Source: PLoS One. 2015 Aug 7;10(8):e0134312. doi: 10.1371/journal.pone.0134312 (PMC4529157; doi:10.1371/journal.pone.0134312)
Supplement: S1 Table — Broth microdilution susceptibility testing was performed with the Merlin Micronaut system; results were interpreted according to Clinical and Laboratory Standards Institute (CLSI) MIC criteria based on CLSI MIC interpretive standards for other non-Enterobacteriaceae and anaerobes [25]. AZM: azithromycin, CIP: ciprofloxacin, CLI: clindamycin, CMP: chloramphenicol, ERY: erythromycin, GEN: gentamicin, MER: meropenem, NAL: nalidixic acid, STR: streptomycin, T/S: trimethoprim/sulfamethoxazole, TEL: telithromycin, TET: tetracycline, R: resistant, I: intermediate susceptible, S: susceptible phenotype, MIC values in mg/L. (DOCX) [file pone.0134312.s002.docx]

**S1 Table. Antimicrobial susceptibility testing of *Streptobacillus moniliformis* strains and of reference strains *Streptobacillus felis* 131000547^T^*, Streptobacillus hongkongensis* DSM 26322^T^ and *Sebaldella termitidis* NCTC 11300^T^*.***

| **Strain no.** | **Strain** | **AZM** | **CIP** | **CLI** | **CMP** | **ERY** | **GEN** | **MER** | **NAL** | **STR** | **T/S** | **TEL** | **TET** |
| --- | --- | --- | --- | --- | --- | --- | --- | --- | --- | --- | --- | --- | --- |
| 1 | DSM 12112^T^ | S | S | S | S | S | S | S | S | S | R | S | S |
|  |  | 0.0625 | =1 | =0.25 | =1 | ≤0.5 | ≤0.125 | =0.25 | =2 | ≤1 | >8/152 | ≤0.125 | ≤0.125 |
| 2 | CIP 55-48 | S | S | S | S | I | I | S | R | I | R | S | S |
|  |  | =0.25 | =0.25 | ≤0.125 | =4 | =16 | =4 | ≤0.0625 | 128 | =8 | >8/152 | =4 | =0.5 |
| 3 | ATCC 27747 | S | S | S | S | S | R | S | R | I | R | S | S |
|  |  | =0.25 | =1 | ≤0.125 | =4 | =4 | =8 | =1 | =64 | =16 | >8/152 | =2 | =1 |
| 4 | NCTC 10773 | S | S | S | S | S | S | S | R | S | R | S | S |
|  |  | =0.25 | =0.5 | ≤0.125 | =2 | =2 | =1 | ≤0.0625 | =64 | =2 | >8/152 | =1 | ≤0.125 |
| 5 | NCTC 11194 | S | I | S | S | I | I | S | R | I | R | S | S |
|  |  | =1 | =2 | ≤0.125 | =4 | =16 | =4 | ≤0.0625 | 128 | =8 | >8/152 | =4 | =0.5 |
| 6 | AHL 370-1 | S | S | S | S | S | S | S | S | I | R | S | S |
|  |  | =0.25 | =1 | ≤0.125 | =1 | ≤0.5 | ≤0.125 | ≤0.0625 | =16 | =4 | =8/152 | =2 | ≤0.125 |
| 7 | IPDH 144/80 | S | S | S | S | S | S | S | R | I | R | S | S |
|  |  | =0.125 | =1 | ≤0.125 | =1 | =2 | =1 | ≤0.0625 | =64 | =8 | >8/152 | =0.5 | ≤0.125 |
| 8 | CIP 81-99 | S | S | S | S | S | S | S | I | I | R | S | S |
|  |  | =0.5 | =1 | ≤0.125 | =4 | =4 | =4 | =0,25 | =32 | =8 | >8/152 | =2 | =0.5 |
| 9 | AHL 370-4 | S | S | S | S | S | S | S | S | I | R | S | S |
|  |  | =1 | =1 | ≤0.125 | ≤0.5 | ≤0.5 | =2 | ≤0.0625 | =16 | =8 | >8/152 | =1 | =0.25 |
| 10 | NCTC 11941 | S | S | S | S | S | S | S | S | S | R | S | S |
|  |  | ≤0.0625 | =1 | ≤0.125 | =2 | =8 | =0.5 | ≤0.0625 | =16 | =2 | =8/152 | =0.25 | ≤0.125 |
| 11 | IPDH 109/83 | S | S | S | S | S | S | S | R | S | R | S | S |
|  |  | =0.5 | =1 | ≤0.125 | =2 | =8 | =2 | ≤0.0625 | =128 | =2 | >8/152 | =2 | =0.25 |
| 12 | ATCC 49567 |  | S | S | S | S | I | S | R | I | R | S | S |
|  |  | =0.5 | =1 | ≤0.125 | =4 | =4 | =4 | ≤0.0625 | =128 | =8 | >8/152 | =2 | =0.5 |
| 13 | Kun 3 (RIVM) | S | S | S | S | S | S | S | R | I | R | S | S |
|  |  | =0.125 | =0,5 | ≤0.125 | =4 | =4 | =2 | =0.5 | =32 | =8 | =8/152 | =0.5 | =0.25 |
| 14 | ATCC 49940 | S | I | S | S | I | I | S | R | I | R | S | S |
|  |  | =0.5 | =2 | ≤0.125 | =4 | =16 | =4 | =0,5 | =64 | =8 | >8/152 | =4 | =0.5 |
| 15 | B10/15 | S | S | S | S | S | S | S | I | I | R | S | S |
|  |  | =0.5 | =0.5 | ≤0.125 | =4 | =4 | =2 | =0.5 | =32 | =8 | >8/152 | =0.5 | =0.25 |
| 16 | A378/1 | S | S | S | S | S | S | S | I | S | R | S | S |
|  |  | =0.25 | =0.5 | ≤0.125 | ≤0.5 | =1 | =1 | ≤0.0625 | =32 | =2 | >8/152 | =0.5 | ≤0.125 |
| 17 | VA11257/2007 | S | S | S | S | S | S | S | I | I | R | S | S |
|  |  | =0.25 | =1 | ≤0.125 | =2 | =2 | =2 | ≤0.0625 | =32 | =16 | >8/152 | =2 | =0.25 |
| 18 | VK105/14 | S | S | S | S | S | S | S | R | R | R | S | S |
|  |  | ≤0.0625 | =0.25 | ≤0.125 | ≤0.5 | ≤0.5 | =1 | ≤0.0625 | =64 | =32 | =8/152 | ≤0.125 | =0.25 |
| 19 | B5/1 | S | I | S | S | S | S | S | R | I | R | S | S |
|  |  | =1 | =2 | ≤0.125 | =4 | =8 | =2 | ≤0.0625 | =64 | =8 | >8/152 | =2 | =0.5 |
| 20 | Marseille | S | S | S | S | S | S | S | S | I | R | S | S |
|  |  | =2 | =2 | ≤0.125 | =4 | =4 | =2 | ≤0.0625 | =64 | =8 | >8/152 | =2 | =0.25 |
| 28 | 131000547^T^ | S | S | S | S | S | S | S | R | I | R | S | S |
|  |  | =0.125 | =1 | ≤0.125 | =4 | =4 | =2 | =0,25 | =32 | =4 | >8/152 | =2 | =0.25 |
| 29 | DSM 26322^T^ | S | S | S | S | S | S | S | S | S | S | S | S |
|  |  | ≤0.0625 | =0.5 | ≤0.125 | ≤0.5 | ≤0.5 | ≤0.125 | ≤0.0625 | ≤1 | ≤1 | ≤0.0625/1.1875 | ≤0.125 | ≤0.125 |
| 30 | NCTC 11300^T^ | R | R | R | R | R | S | I | R | R | R | R | I |
|  |  | >8 | >8 | =8 | =32 | >64 | =0.5 | =8 | >128 | >64 | >8/152 | >16 | =8 |

Broth microdilution susceptibility testing was performed with the Merlin Micronaut system; results were interpreted according to Clinical and Laboratory Standards Institute (CLSI) MIC criteria based on CLSI MIC interpretive standards for other non-*Enterobacteriaceae* and anaerobes [[18](#_ENREF_18)]. AZM: azithromycin, CIP: ciprofloxacin, CLI: clindamycin, CMP: chloramphenicol, ERY: erythromycin, GEN: gentamicin, MER: meropenem, NAL: nalidixic acid, STR: streptomycin, T/S: trimethoprim/sulfamethoxazole, TEL: telithromycin, TET: tetracycline, R: resistant, I: intermediate susceptible, S: susceptible phenotype, MIC values in mg/L
