# Supplementary material for: Phenotypic and Genotypic Characteristics of Members of the Genus Streptobacillus
Source: PLoS One. 2015 Aug 7;10(8):e0134312. doi: 10.1371/journal.pone.0134312 (PMC4529157; doi:10.1371/journal.pone.0134312)
Supplement: S2 Table — (DOCX) [file pone.0134312.s003.docx]

**S2 Table. Guanin/cytosin (G/C) contents of *Streptobacillus* spp. isolates and average nucleotide identity (ANI) of all strains under study to *S. moniliformis* type strain DSM 12112^T^.**

| **Strain designation** | **Species** | **G/C content (%)** | **ANI to DSM 12112^T^ (reciprocal)** |
| --- | --- | --- | --- |
| DSM 12112^T^ | *Streptobacillus moniliformis* | 26.3 | 100 (100) |
| CIP 55-48 | *Streptobacillus moniliformis* | 26.1 | 99.13 (98.90) |
| ATCC 27747 | *Streptobacillus moniliformis* | 26.1 | 98.61 (98.92) |
| NCTC 10773 | *Streptobacillus moniliformis* | 28.9 | 98.96 (98.21) |
| NCTC 11194 | *Streptobacillus moniliformis* | 26.1 | 99.30 (98.59) |
| AHL 370-1 | *Streptobacillus* sp. | 26.1 | 88.43 (87.89) |
| IPDH 144/80 | *Streptobacillus moniliformis* | 26.1 | 98.99 (99.15) |
| CIP 81-99 | *Streptobacillus moniliformis* | 26.4 | 99.16 (99.39) |
| AHL 370-4 | *Streptobacillus moniliformis* | 25.9 | 98.60 (99.23) |
| NCTC 11941 | *Streptobacillus moniliformis* | 25.7 | 99.27 (98.33) |
| IPDH 109/83 | *Streptobacillus moniliformis* | 26.1 | 99.00 (99.27) |
| ATCC 49567 | *Streptobacillus moniliformis* | 26.1 | 99.00 (99.35) |
| Kun 3 | *Streptobacillus moniliformis* | 25.9 | 98.69 (99.38) |
| ATCC 49940 | *Streptobacillus moniliformis* | 25.9 | 98.93 (99.09) |
| B10/15 | *Streptobacillus moniliformis* | 26.2 | 98.99 (99.21) |
| A378-1 | *Streptobacillus moniliformis* | 26.0 | 98.78 (99.19) |
| VA11257/2007 | *Streptobacillus moniliformis* | 26.1 | 98.77 (98.77) |
| VK105/14 | *Streptobacillus moniliformis* | 26.2 | 99.15 (99.41) |
| B5/1 | *Streptobacillus moniliformis* | 25.8 | 98.51 (98.99) |
| Marseille | *Streptobacillus moniliformis* | 26.1 | 99.15 (98.17) |
| IKC1 | *Streptobacillus moniliformis* | 26.0 | 99.21 (98.86) |
| IKC5 | *Streptobacillus moniliformis* | 26.2 | 98.65 (98.92) |
| IKB1 | *Streptobacillus moniliformis* | 25.9 | 99.14 (99.07) |
| TSD4 | *Streptobacillus moniliformis* | 26.0 | 98.90 (99.10) |
| OGS16 | *Streptobacillus* sp. | 25.9 | 89.10 (89.87) |
| KWG2 | *Streptobacillus* sp. | 26.4 | 88.21 (88.57) |
| KWG24 | *Streptobacillus* sp. | 26.3 | 88.25 (88.66) |
| 131000547^T^ | *Streptobacillus felis* | 26.4 | 82.00 (82.02) |
| DSM 26322^T^ | *Streptobacillus hongkongensis* | 26.1 | 74.04 (75.03) |
| NCTC 11300^T^ | *Sebaldella termitidis* | 33.4 | 67.45 (66.73) |
